# Protein Corona Gold Nanoparticles Fingerprinting Reveals a Profile of Blood Coagulation Proteins in the Serum of HER2-Overexpressing Breast Cancer Patients

**DOI:** 10.3390/ijms21228449

**Published:** 2020-11-10

**Authors:** María del Pilar Chantada-Vázquez, Antonio Castro López, María García-Vence, Benigno Acea-Nebril, Susana B. Bravo, Cristina Núñez

**Affiliations:** 1 Research Unit, Lucus Augusti University Hospital (HULA), Servizo Galego de Saúde (SERGAS), 27002 Lugo, Spain; mariadelpilarchantadavazquez@gmail.com; 2 Proteomic Unit, Health Research Institute of Santiago de Compostela (IDIS), University Clinical Hospital of Santiago de Compostela (CHUS), 15706 Santiago de Compostela, Spain; mariagarve@outlook.es; 3 Breast Unit, Hospital Universitario Lucus Augusti (HULA), Servizo Galego de Saúde (SERGAS), 27002 Lugo, Spain; Antonio.Castro.Lopez@sergas.es; 4 Department of Surgery, Breast Unit, Complexo Hospitalario Universitario A Coruña (CHUAC), Servizo Galego de Saúde (SERGAS), 15006 A Coruña, Spain; Benigno.Acea.Nebril@sergas.es

**Keywords:** protein corona (PC), gold nanoparticles (AuNPs), breast cancer (BC), fingerprinting, SWATH-MS, HER2+

## Abstract

Breast cancer (BC) is a molecularly heterogeneous disease that encompasses five major molecular subtypes (luminal A (LA), luminal B HER2 negative (LB-), luminal B HER2 positive (LB+), HER2 positive (HER2+) and triple negative breast cancer (TNBC)). BC treatment mainly depends on the identification of the specific subtype. Despite the correct identification, therapies could fail in some patients. Thus, further insights into the genetic and molecular status of the different BC subtypes could be very useful to improve the response of BC patients to the range of available therapies. In this way, we used gold nanoparticles (AuNPs, 12.96 ± 0.72 nm) as a scavenging tool in combination with Sequential Window Acquisition of All Theoretical Mass Spectra (SWATH-MS) to quantitatively analyze the serum proteome alterations in the different breast cancer intrinsic subtypes. The differentially regulated proteins specific of each subtype were further analyzed with the bioinformatic tools STRING and PANTHER to identify the major molecular function, biological processes, cellular origin, protein class and biological pathways altered due to the heterogeneity in proteome of the different BC subtypes. Importantly, a profile of blood coagulation proteins was identified in the serum of HER2-overexpressing BC patients.

## 1. Introduction

Breast cancer (BC) is a heterogeneous disease that presents a wide variety of molecular and clinical characteristics, as well as variability in clinical progression [1]. For the treatment choice, patients are classified according to intrinsic biological subtypes within the BC spectrum, using clinical-pathological criteria, i.e., the recognition of amplification and/or overexpression of the human epidermal growth factor receptor 2 (HER2) oncogene, the immunohistochemical classification of the estrogen receptor (ER) and the progesterone receptor (PR) and Ki-67 labeling index [2]. This classification allows for a more personalized approach to medical treatments, with favorable results. However, despite that almost 10–15% of these patients still experience local or distant recurrences in the first five years from diagnosis [3]. Particularly, HER2-positive BC, defined by the overexpression of HER2 protein, represents 15–20% of BC cases [4,5] and is correlated with poor prognosis, high rates of recurrence and short survival [6].

Classification of BC might be markedly improved if new biomarkers identified with the use of high-throughput “omics” approaches could support diagnosis based on histopathological patterns [7,8,9]. Nanomaterials have been introduced into the field of proteomics to establish a new and rapidly evolving research area termed nanoproteomics [10].

It is well known that the dispersion of a nanomaterial in physiological fluid results in the formation of a protein shell named “protein corona” (PC). PC varies depending on the characteristics of the biological media, the physical (size, shape, curvature) and chemical properties (composition, surface charge/chemistry and hydrophobicity/hydrophilicity) of the nanomaterial and the incubation time [11]. Disease-associated biomarkers comprise less than 1% of serum proteins. In this way, through the formation of the PC, nanoparticles could act as sorbent materials for the enrichment of low-abundance peptides/proteins presented in serum samples before the biomarker identification by mass spectrometry (MS) analysis [12,13,14]. Importantly, otherwise undetectable changes in protein concentration at an early stage of the disease (as breast cancer), after any treatment (chemotherapy, immunotherapy) or surgery could be detected analyzing the PC composition [15]. Thus, characterization of the PC around NPs offers distinct advantages over sole proteomic approaches and increases the success of identifying molecular targets [16].

**Figure 1:**
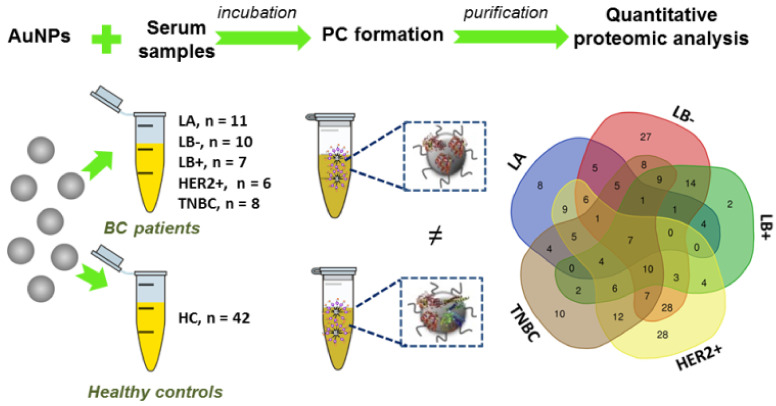
Simplified representation of the experimental procedure. AuNPs (12.96 ± 0.72 nm) were incubated ex vivo with human serum samples obtained from HC (*n* = 42) and BC patients (*n* = 42). Ex vivo corona-coated AuNPs were recovered and purified from unbound proteins by centrifugation. The formed PCs were quantitatively characterized, analyzed by SWATH-MS and compared between the groups.

Particularly, AuNPs present some properties to be used as suitable sorbent nanomaterials: high surface-area-to-volume ratio, colloidal stability and the ability to conjugate with biomolecules [17]. Here, the interaction of AuNPs (12.96 ± 0.72 nm) with the sera of disease-free women (healthy controls, HC) (*n* = 42) and BC patients (*n* = 42) allowed the pre-concentration of the low-abundance proteins thorough the PC formation. Then, an exhaustive quantitative analysis of the PCs by SWATH-MS was carried out to identify novel molecular targets associated to the different BC intrinsic subtypes (see Figure 1).

## 2. Results

### 2.1. Incubation of AuNPs (12.96 ± 0.72 nm) with Human Serum Samples: Ex Vivo Protein Corona Formation and Characterization

**Figure 2:**
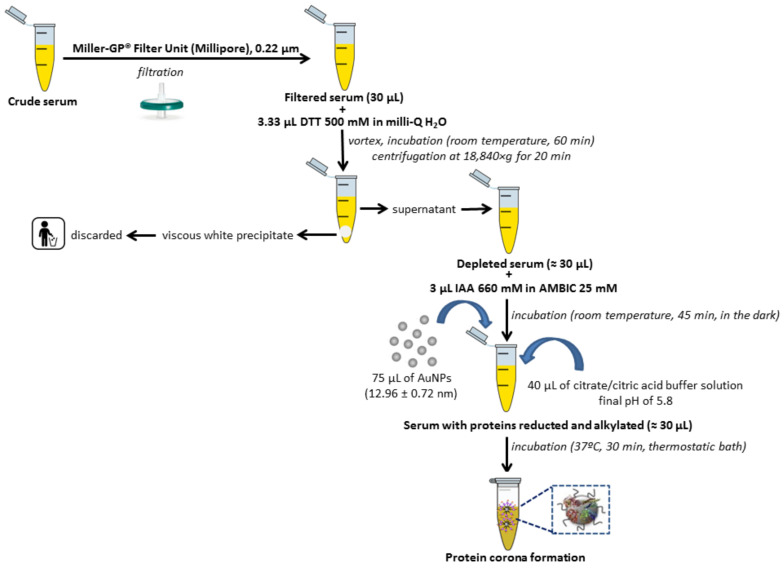
Flowchart depicting serum samples pretreatment and protein corona formation.

Human serum samples from *n* = 42 healthy controls (HC) and *n* = 42 breast cancer (BC) patients (*n* = 42) were recruited, handled and analyzed in the same way as further detailed in Figure 2. The group of BC patients was divided into the following biological subtypes: *n* = 11 patients with the luminal A (LA) subtype, *n* = 10 patients with the luminal B HER2 negative (LB-) subtype, *n* = 7 patients with the luminal B HER2 positive (LB+) subtype, *n* = 6 patients with the HER2 positive (HER2+) subtype and *n* = 8 patients with the triple negative breast cancer (TNBC) subtype. Patient clinical characteristics are summarized in Table S1.

AuNPs with a size of 12.96 ± 0.72 nm were prepared by a chemical reduction method [12,13,14]. As Figure 2 shows, proteins presented in serum samples (×2) were chemically reduced with dithiothreitol (DTT) and alkylated with iodoacetamide (IAA) before their ex vivo incubation with AuNPs (12.96 ± 0.72 nm) to get the formation of the PCs [12,13,14].

**Figure 3:**
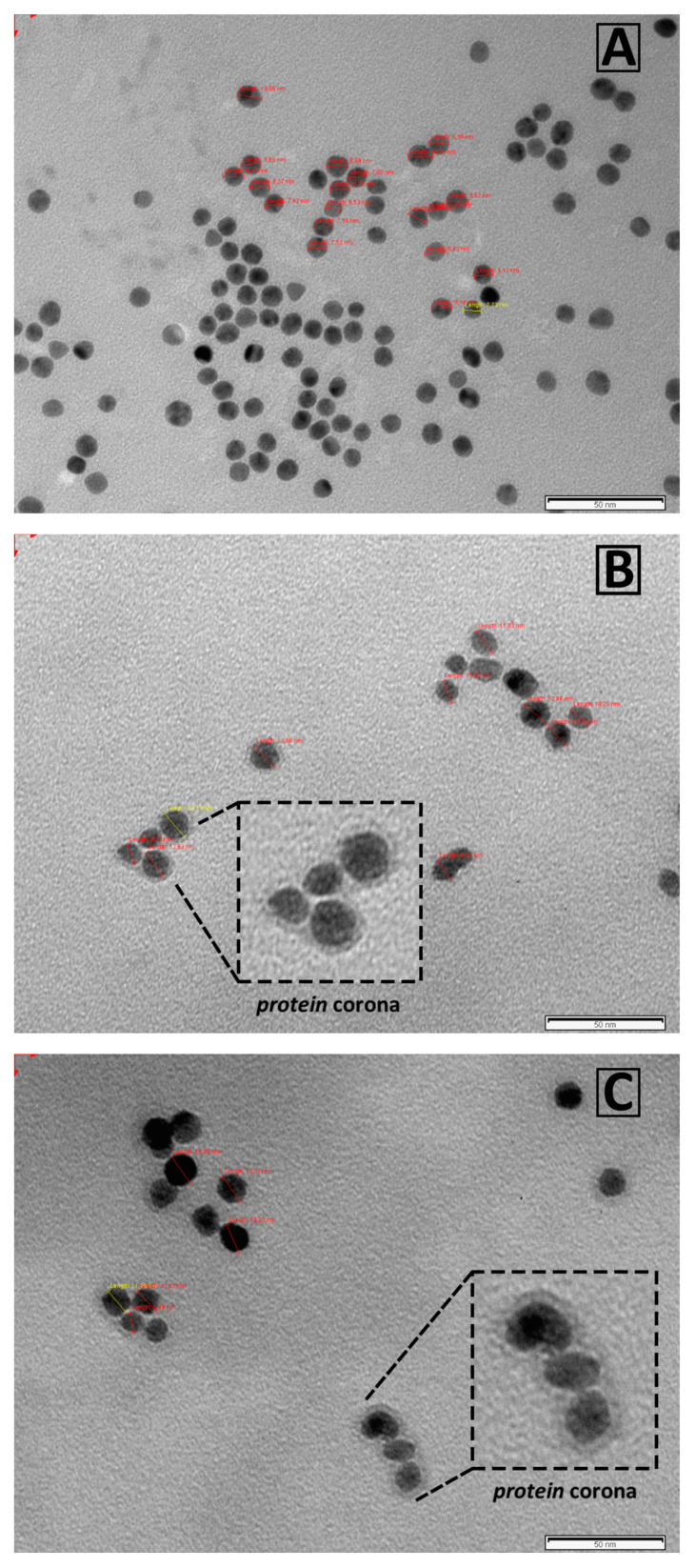
Negative stain TEM imaging of bare (**A**) and protein corona-coated AuNPs, recovered post-incubation with human serum obtained from HC (**B**) and BC patients (**C**). All scale bars are 50 nm.

After the ex vivo incubation of AuNPs with human serum samples of HC (*n* = 42) and BC patients (*n* = 42), the resultant protein corona-coated AuNPs were centrifugated and structurally characterized by dynamic light scattering (DLS) and negative stain transmission electron microscopy (TEM). DLS measurements showed that the interaction of serum proteins with the surface of AuNPs resulted in an increase of the size of the AuNPs, from 12.96 ± 0.72 to 17.33 ± 1.55 (HC) and 17.13 ± 1.53 nm (BC) (see Table S2). Probably, the preferential interaction of positively charged proteins with the AuNPs surface promoted the increase of the mean particle surface charge from −38.3 (bare AuNPs) to −30.5 (HC) and −30.3 mV (BC) [18,19]. TEM imaging revealed a well-dispersed nanoparticles population corroborating the PC formation around AuNPs (see Figure 3).

### 2.2. Quantitative Analysis of the Protein Corona-Coated AuNPs by SWATH-MS

Corona proteins associated with AuNPs were separated by sodium dodecyl sulfate–polyacrylamide gel electrophoresis (SDS-PAGE). After a staining step, gels were processed following the method described in Section 4.5. The resulting peptides were then quantitatively analyzed by the emerging proteomic platform for label-free quantification SWATH-MS.

The comparison of the protein patterns of the ex vivo formed PCs allowed the identification of the differentially expressed proteins between HC and the different BC subtypes. The results were filtered to present a *p*-value ≤ 0.05, and, interestingly, *n* = 60 proteins were found to be differentially expressed, of which *n* = 42 were upregulated and *n* = 18 downregulated in BC patients for the LA subtype; *n* = 132 were found to be differentially expressed (*n* = 100 upregulated and *n* = 32 downregulated) for the LB- subtype; *n* = 67 proteins were found to be differentially expressed (*n* = 59 upregulated and *n* = 8 downregulated) for the LB+ subtype; *n* = 130 proteins were found to be differentially expressed (*n* = 95 upregulated and *n* = 35 downregulated) for the HER2+ subtype; and *n* = 91 proteins were found to be differentially expressed (*n* = 87 upregulated and *n* = 4 downregulated) for the TNBC subtype (see Table 1). The full list of candidate protein biomarkers identified to be upregulated or downregulated in each different BC subtypes in comparison to healthy controls (HC) with the fold-change values is shown in Table S3.

**Figure 4:**
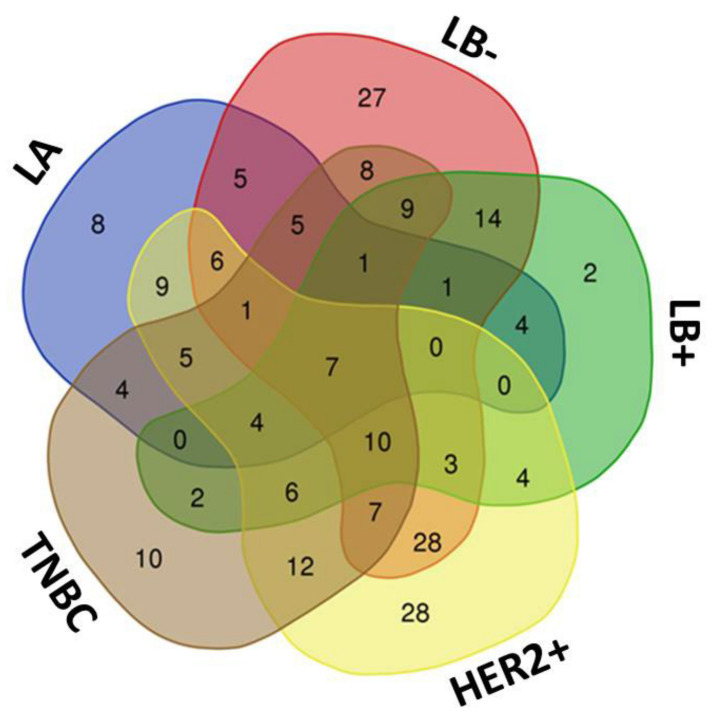
Venn diagram showing the number of shared and specific (or unique) deregulated proteins identified in the PCs formed after the interaction of AuNPs (12.96 ± 0.72 nm) with serum samples of the different BC subtypes (LA, LB-, LB+, HER2+ and TNBC).

The Venn diagram of statistically significant (up- and down)regulated proteins shows that seven proteins were found to be commonly altered in all BC subtypes (see Figure 4): apolipoprotein C-III (APOC3), c-reactive protein (CRP), hemoglobin subunit beta (HBB), immunoglobulin heavy variable 3–49 (IGHV3–49), serum amyloid A-4 protein (SAA4), serum amyloid P-component (APCS) and serotransferrin (TF) (Tables S4 and S5).

As mentioned above, subtype-specific unique proteins were also identified using SWATH-MS (see Figure 4 and Table 2). Overall, *n* = 8 proteins were specifically associated in LA (of which *n* = 4 were upregulated and *n* = 4 showed downregulation). For LB-, *n* = 27 proteins were found to be specific to this subtype (*n* = 25 with increased expression and *n* = 2 with decreased expression). In the LB+ subtype, only *n* = 2 specific proteins were found to be upregulated. In the HER2+ subtype, *n* = 28 specific proteins were found (*n* = 23 upregulated and *n* = 5 downregulated). The TNBC subtype comprised of *n* = 10 specific proteins (*n* = 9 upregulated and *n* = 1 downregulated).

### 2.3. Functional Pathway and Network Analysis for Subtype Specific Breast Cancer

**Figure 5:**
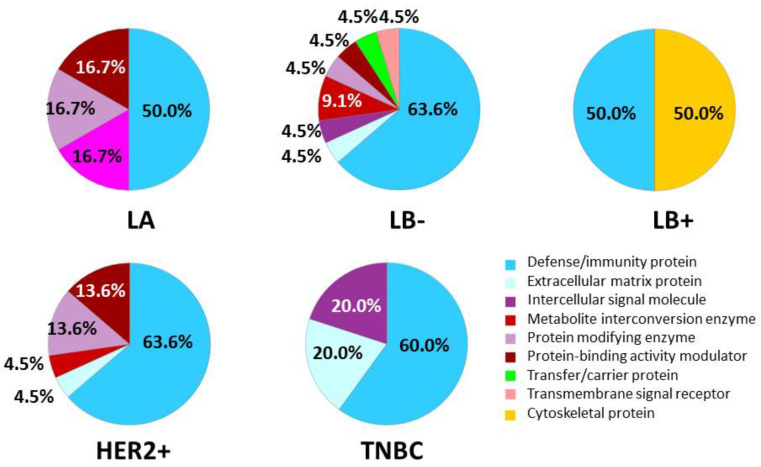
Classification according to the protein class of the differentially regulated proteins specific to each of the five subtypes of BC found in the ex vivo formed coronas analyzed with the PANTHER database.

The differentially regulated proteins specific to each of the five subtypes of BC found in the ex vivo formed coronas were analyzed with the PANTHER [20] tool to identify the major molecular function (Figure S1), biological processes (Figure S2), cellular origin (Figure S3), protein class (Figure 5) and biological pathways (Figure S4) altered due to the heterogeneity in proteome of the different BC subtypes.

Molecular functions of the differentially regulated proteins specific to each of the five subtypes of BC were found to be associated with binding, catalytic activity, molecular regulation and transportation (Figure S1). Furthermore, with the exception of the specific proteins identified in the LB+ subtype, the majority of profiled proteins were of extracellular origin (Figure S3).

During the past decade, insight has been gained about the role of the immunological response in the BC disease process [21] and the possible use of immunological parameters in the prognosis of BC [22]. The PANTHER classification according to their protein class revealed that most of the differential proteins belong to defense/immunity (Figure 5). In the present work, from the 75 specific proteins identified for the different BC subtypes (*n* = 8 in LA; *n* = 27 in LB-; *n* = 2 in LB+; *n* = 28 in HE; and *n* = 10 in TNBC), 34 proteins were immunoglobulins (*n* = 3 in LA; *n* = 14 in LB-; *n* = 1 in LB+; *n* = 13 in HER2+; and *n* = 3 in TNBC) (see Table 2). Previous works also found that serum immunoglobulin levels were related to the disease stage and tumor load in BC patients [23].

Immune cell activation was also shown to be an altered pathway in BC which was enriched only for the LA subtype in the present study (Figure S4). Immunoglobulin heavy constant mu (IGHM) was downregulated for B cell activation in LA, indicating that antibody-mediated immune response was implicated in this subtype. Probably, the tumor alters the immune system mechanism to suppress the B cell activation promoting this downregulation.

Complement activation is an important factor of innate immunity and a defense system against infecting pathogens. Furthermore, complement activation also participates in the adaptive immune response. Particularly in BC, complement activation contributes to cancer progression [24]. In the present work, three complement system components implicated in the innate immune response were identified in the PC for the different BC subtypes: complement component C8 beta chain (C8B) for LA, complement C5 (C5) for HER2+ and complement C2 (C2) for TNBC (see Table 2). Previous studies also found deregulation of some of these complement system components in the sera of BC patients [25,26].

Other deregulated proteins which may play a role in the innate immune system were complement C1r subcomponent-like protein (C1RL) and complement factor H-related protein 2 (CFHR2) (both upregulated) in the LA subtype; complement C1s subcomponent (C1S) (downregulated) in the LB- subtype; plastin-2 (LCP1) (upregulated) in the LB+ subtype; cysteine-rich secretory protein 3 (CRISP3) (upregulated) and N-acetylmuramoyl-L-alanine amidase (PGLYRP2) (downregulated) in the HER2+ subtype; and keratin, type II cytoskeletal 1 (KRT1) (upregulated) in the TNBC subtype (see Table 2). Particularly, LCP1 plays a role in the activation of T-cells and its exosomal release by breast cancer cells was found to facilitate metastatic bone osteolysis [27].

After the analysis of the protein corona, HER2+ subtype was enriched with coagulation factor V (F5), coagulation factor XII (F12) and plasma kallikrein (KLKB1) (upregulated), while alpha-1-antitrypsin (SERPINA1), trypsin-1 (PRSS1) and antithrombin-III (SERPINC1) (downregulated) for blood coagulation pathway (see Table 2 and Table 3, and Figure S4).

Platelets, as small cell fragments, are not only important coagulation-related factors, also play a vital role in tumor progression [28]. Particularly, platelet factor 4 (PF4) or CXCL4, a member of CXC chemokine family, acts as an angiogenesis inhibitor which may contribute to prevent tumor metastasis [29]. In the present work, it was observed that the inflammation mediated by chemokine and cytokine signaling pathway was enriched specifically for LB- subtype with the platelet factor 4 variant (PF4V1) being downregulated (Figure S4). Another protein of the family of platelets, the platelet basic protein (PPBP), was upregulated in the TNBC subtype (see Table 2).

The gonadotropin-releasing hormone receptor pathway was found to be enriched with adiponectin (ADIPOQ) (upregulated) and voltage-dependent L-type calcium channel subunit alpha-1F (CACNA1F) (upregulated) in the HER2+ and TNBC subtypes, respectively (Figure S4).

In the present work, a group of proteins implicated in the combination and transportation of lipids, apolipoproteins, were also found to be deregulated in the LB- (apolipoprotein B-100 (APOB), apolipoprotein D (APOD) and phospholipid transfer protein (PLTP); upregulated), HER2+ (apolipoprotein F (APOF); downregulated) and TNBC subtypes (apolipoprotein E (APOE); upregulated) (see Table 2).

Another family of potential molecular targets that was found to be deregulated in the present study includes some glycoproteins: lysosome-associated membrane glycoprotein 2 (LAMP2) (upregulated) in the LA subtype; platelet glycoprotein Ib alpha chain (GP1BA) and basement membrane-specific heparan sulfate proteoglycan core protein (HSPG2) (upregulated) in the LB- subtype; EGF-containing fibulin-like extracellular matrix protein 1 (EFEMP1) and selenoprotein P (SELENOP) (upregulated) in the HER2+ subtype; and CD5 antigen-like (CD5L) (downregulated) in the TNBC subtype (see Table 2).

While some proteins with an enzymatic functionality such as biotinidase (BTD), serum paraoxonase/lactonase 3 (PON3), L-lactate dehydrogenase B chain (LDHB) and alpha-mannosidase 2 (MAN2A1) were found to be the upregulated in LB- subtype, protein Z-dependent protease inhibitor (SERPINA10) were downregulated in the LA subtype (see Table 2).

## 3. Discussion

Nowadays, the different breast cancer intrinsic subtypes (LA, LB-, LB+, HER2+ and TNBC) guide the therapy selection [2]. In some patients, therapies could fail for different reasons such as cancer recurrence, therapy resistance and/or metastasis. Thus, therapy response in breast cancer patients could be improved with the study of the molecular alterations at the subtype level.

The application of different omics approaches to deep insight the different BC subtypes allowed the refinement of the complexity of tumor heterogeneity. In this way, a variety of quantitative proteomic studies were developed to identify potential signatures for breast cancer using clinical samples such as saliva, the ductal lavage fluids, nipple aspirate fluids (NAFs), urine and tissue [30]. Importantly, blood may be a suitable sample source for studying the proteomic deregulation in the different BC subtypes with minimally invasive collection procedures. Thus, the analysis of blood-based markers at the subtype level in biological fluids such as plasma and serum allowed finding different protein markers related with the tumor microenvironment and the subtype-specific changes. Following this research line, the proteomic alterations in blood plasma [7] and blood serum [8] of BC subtypes were explored.

Potential biomarkers are presented in very low concentrations in blood (less than 1% of blood proteins). Thus, the isolation and enrichment of low-abundance peptides/proteins from complex mixtures is a mandatory step in the proteomic biomarkers pipeline, and nanoparticles represent an ideal alternative [31].

In the present work, an exhaustive quantitative analysis of the PCs formed around AuNPs after their incubation in serum samples was developed by SWATH-MS to identify novel molecular targets associated to the different BC intrinsic subtypes (see Figure 1).

Seven proteins were found to be commonly altered in all BC subtypes, namely APOC3, CRP, HBB, IGHV3–49, SAA4, APCS and TR. CRP and SAA4 are acute-phase proteins (APPs), a class of proteins whose serum concentrations increase or decrease in response to inflammation. Particularly, a significant association of state of inflammation with stage of BC was previously described [32]. A recent study found that elevated serum levels of CRP were associated considerably with a high risk of BC and poor outcome, including metastasis and recurrence [33]. In addition, the concentrations of SAA4 increased gradually with tumor progression and the severity of BC stages [34]. Thus, CRP and SAA4 may be good candidate markers for the staging and prognosis of BC. The expression of HBB and TR, members of the globin family was also found to be associated with BC cells aggressiveness and poor prognosis, indicating HBB and TR as novel biomarker for BC progression [35,36].

On the other hand, several studies point out that blood coagulation proteins develop an important role in tumor progression [37]. These works discussed the impact of the activation of the blood clotting cascade on primary tumor growth [38], tumor metastasis and cancer-associated thrombosis [39] and antitumor therapies that target blood-coagulation-associated proteins [40].

In the particular case of BC, different reports support blood coagulation proteins as an important patient factor that facilitates the metastatic potential [41]. Particularly, metastatic patients exhibited significantly higher D-dimer values when compared with early breast cancer patients [42]. Furthermore, high plasma fibrinogen was found to be correlated with poor response to trastuzumab treatment in HER2 positive BC patients [43] and circulating levels of factor VIII (FVIII) were significantly associated with axillary lymph node involvement, number of metastatic nodes and HER2 status [44]. These studies support that the measurement of some coagulation-related biomarkers could provide additional data for the evaluation of HER2 positive BC patients’ prognosis and could be novel molecular targets.

The present quantitative proteomic analysis revealed a profile of blood coagulation proteins for the HER2+ subtype, namely F5, F12 and KLKB1 (upregulated), and SERPINA1, PRSS1 and SERPINC1) (downregulated). While F5 is expressed in tumors and indicates favorable outcome in aggressive BC [45], F12 is involved in the pathogenesis of thrombosis through the induction and amplification of thrombin generation [46].

KLKB1 (up), SERPINA1 (down) and SERPINC1 (down) are serine proteases. Particularly, SERPINA1 and SERPINC1 are serine proteases inhibitors (serpins) which belong to the protease inhibitor family. Members of the kallikrein family, such as KLKB, were also found to be deregulated during malignant transformation [47]. Nevertheless, the variations in expression (downregulation/upregulation), activation and secretion are not substantial enough to consider them as suitable biomarkers for follow-up disease progression. SERPINA1 is synthesized and released by tumor cells and plays major roles in physiologic and pathologic processes such as angiogenesis, tumor invasion and metastasis [48]. It was found that that high expression of SERPINA1 could be predictive for a better clinical outcome of ER+ and ER+/HER2+ patients. Thus, SERPINA1 was found to be a direct ER target gene and a predictor of survival in BC patients [49]. SERPINC1, an antithrombin, develops an important role as an inhibitor of the coagulation cascade. Furthermore, SERPINC1 also functions as an anti-angiogenic, anti-inflammatory, anti-viral and anti-apoptotic protein. The mechanism by which antithrombin controls invasion, tumor migration and angiogenesis is by inhibition of enteropeptidase. This inhibition showed to be a double anti-tumor effect through producing an anti-angiogenic molecule and inhibiting a protease implicated in metastasis [50].

In the present work, the gonadotropin-releasing hormone receptor pathway was found to be enriched with adiponectin (ADIPOQ) in the HER2+ subtype. Although different studies reported controversial findings in the association between ADIPOQ and BC, a recent meta-analysis suggests that low serum adiponectin concentration may be associated with an increased BC risk in premenopausal and postmenopausal women [51]. A negative correlation has been also demonstrated between ADIPOQ levels and tumor size and grade. Interestingly, the correlation between ADIPOQ and BC seems to be more prominent in estrogen-negative and progesterone-negative BC [52]. Therefore, it seems there may be a set group of BC patients that are most susceptible to the effects of ADIPOQ and would benefit most from a potential treatment. ADIPOQ may serve as a biomarker of BC risk and help to identify subjects at high risk for BC development.

Numerous research articles have accumulated solid evidence that lipoproteins are closely related to various types of tumorigenesis, as BC [53]. Apolipoproteins in the blood transfer lipids to cancer cells to provide energy for cancer cell proliferation and invasion. Apolipoproteins also function as important factors in cellular signal transduction. In the present work, different apolipoproteins were found to be deregulated in the different BC subtypes. Particularly, APOB, APOD and APOE in serum were found to function as a risk factor for BC, being APOD and APOB involved in BC metastasis [54]. Particularly, a recent study found that apolipoprotein B is a risk factor for development of intraocular metastasis (IOM) in patients with BC [55].

Within the group of glycoproteins found to be deregulated in the present study, it was found that LAMP2 overexpression in breast tumors promotes cancer cell survival via chaperone-mediated autophagy (CMA) [56]. Thus, inhibiting CMA activity in breast tumor cells (with a chemotherapeutic drug, for example) can be exploited as a potential therapeutic application in the treatment of BC. HSPG2, also known as perlecan, is a heavily glycosylated protein component of the extra-cellular matrix (ECM) that plays essential roles in tumor vascularization, that is closely related to tumor growth and metastasis [57]. Although HSPG2 expression in BC has not been examined in detail, a recent study investigated the expression of HSPG2 in human TNBC and the ability of anti-HSPG2 antibodies to specifically target and inhibit tumor growth in a mouse xenograft model [58], showing that HSPG2 is a promising therapeutic target in TNBC. EFEMP1, also known as fibulin 3, may have a potential cancer-promoting function in BC [59]. EFEMP1 expression decreases during BC progression, with low EFEMP1 levels correlating with a poorer prognosis. Functionally, high EFEMP1 levels inhibited TGF-β-induced EMT, migration, invasion and endothelial permeability, while loss of EFEMP1 expression/function promoted these TGF-β-mediated effects. Further, restoring EFEMP1 expression in breast cancer cells inhibited TGF-β signaling, breast cancer cell EMT, invasion and metastasis in vivo. Although the role of CD5L in the oncogenesis of BC is not fully understood, a recent work found that CD5L is upregulated in hepatocellular carcinoma and promotes liver cancer cell proliferation and antiapoptotic responses [60].

The enzyme LDHB, which was found to be upregulated in the LB- subtype, may help identify breast cancers most likely to respond to neoadjuvant chemotherapy as well as those with the highest risk of relapse that may benefit from additional adjuvant therapy [61].

All these novel molecular targets found in the serum of BC patients could detect a missing invasion, be performed in ambulatory settings, be repeatedly checked, and be applicable for BC diagnosis, the assessment of prognosis and selection of treatment.

Importantly, further insights exploring the deregulated blood coagulation proteins as potential effective prognosis biomarkers and targets for novel therapeutic approaches could have a great impact in the management of HER2-overexpressing BC patients.

## 4. Materials and Methods

### 4.1. Chemicals

Acrylamide/bis-acrylamide 30% solution (37.5:1), *β*-mercaptoethanol (molecular biology grade), Coomassie Brilliant Blue R250 (CBB), DL-dithiothreitol (HSCH_2_CH(OH)CH(OH)CH_2_SH, 99%) (DTT), glycerol (HOCH_2_CH(OH)CH_2_OH, 86–88%), iodoacetamide (IAA, ICH_2_CONH_2_, 99%), sodium citrate tribasic dehydrate (HOC(COONa)(CH_2_COONa)_2_·2H_2_O, 99%), sodium carbonate (Na_2_CO_3_, 99%), tris-base (NH_2_C(CH_2_OH)_3_), trifluoroacetic acid (CF_3_COOH, 99%), trypsin from bovine pancreas and the Sigma Marker wide range 6.5–200 kDa were purchased from Sigma-Aldrich. Formaldehyde for molecular biology (36.5–38% in H_2_O) and sodium dodecyl sulfate (SDS, CH_3_(CH_2_)_11_SO_4_Na) were purchased from Panreac. Bromophenol-blue was purchased from Riedel-de Haen. Hydrogen tetrachloroaurate (III) hydrate (HAuCl_4_·xH_2_O) (99.9%-Au) (49% Au) at 10% *w*/*v* was purchased from Strem Chemicals. Ammoniumbicarbonate (AMBIC, NH_4_HCO_3_, 99.5%) and formic acid (HCOOH, 95%) were purchased from Fluka.

### 4.2. Biological Samples

Blood samples were collected from *n* = 42 newly diagnosed BC patients with the five different breast cancer subtypes: *n* = 11 patients with the luminal A subtype (ER positive, HER2 negative, Ki-67 low, and PR high), *n* = 10 patients with the luminal B-HER2 negative subtype (ER positive, HER2 negative, and either Ki-67 high or PR low), *n* = 7 patients with the luminal B-HER2 positive subtype (ER positive, HER2 overexpressed or amplified, any Ki-67 and any PR), *n* = 6 patients with the HER2 positive subtype (HER2 over-expressed or amplified, ER and PR absent) and *n* = 8 patients with the triple negative subtype (ER and PR absent and HER2 negative).

Blood samples were also collected from *n* = 42 age-matched and gender-matched healthy women (controls). In all cases, venous blood samples were collected in VACUETTE^®^ Serum Clot Activator Tubes (10 mL).

The experiment was conducted in conformity with the declaration of Helsinki and approved by the Clinical Research Ethics Committees (CEIC) of Galicia (Spain) with approval number 2017-021. All participants from Lucus Augusti University Hospital (Spain) gave written informed consent prior to their participation.

### 4.3. Synthesis of Citrate-Gold Nanoparticles (AuNPs, 12.96 ± 0.72 nm)

Colloidal AuNPs with a size of 12.96 ± 0.72 nm were prepared by chemical reduction method as per the protocol developed previously [12,13,14]. In short, sodium citrate tribasic solution (0.075% *w*/*v*) was dissolved in 60 mL warm distilled water under constant magnetic stirring. To this, 54 μL of 10% *w*/*v* of hydrogen tetrachloroaurate (III) hydrate solution was then added drop-wise and the reaction was heated to 100 °C under constant magnetic stirring. Reaction was allowed to proceed further the color of the solution changes from yellow to deep red indicating the reduction of Au^3+^ to Au^0^, which spontaneously aggregates to form colloidal AuNPs. This colloidal dispersion of AuNPs was cooled to room temperature and preserved at 4 °C for further analysis.

### 4.4. Incubation of AuNPs with Human Serum Samples: Ex Vivo Protein Corona Formation

Firstly, collected blood samples were allowed to clot for 15 min. After that, samples were centrifuged for 5 min at 4 °C and 1800× *g*. Resultant serum were transferred to sterile cryovials, frozen and stored at −80 °C until further use at Research Unit, Lucus Augusti University Hospital (HULA). The formation of the ex vivo PC was achieved following the steps shown in Figure 2 [12,13,14]. After that, unbound serum proteins from the surface of AuNPs were removed by centrifugation at 18,840× *g* for 30 min.

### 4.5. Characterization of Colloidal AuNPs

The morphology of the AuNPs was investigated by transmission electron microscopy (TEM) with a JEM 1011, JEOL instrument. The size and ζ-potentials of colloidal AuNPs were measured (3 determinations per sample) with a Malvern Zetasizer Nano ZS instrument at 25 °C.

Protein quantification and protein separation by SDS-PAGE were carried out with the use of a Qubit™ 4 Quantitation Starter Kit (Thermo Fisher Scientific) and a PowerPac^TM^ Basic Power Supply (Bio-Rad), respectively.

### 4.6. Separation and Digestion of the Proteins Presented in the Corona-Coated AuNPs

**Figure 6:**
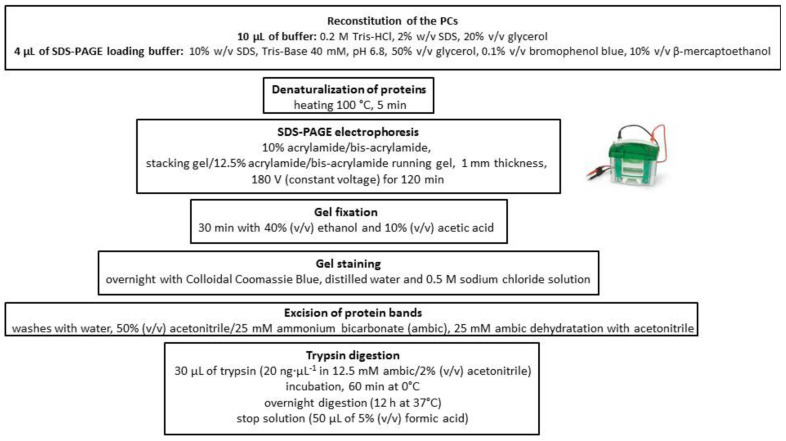
Flowchart depicting the separation and digestion of the corona proteins associated with AuNPs.

Corona proteins associated with AuNPs were separated by sodium dodecyl sulfate–polyacrylamide gel electrophoresis (SDS-PAGE) and digested following the scheme represented in Figure 6.

The digestion was stopped with the addition of 50 μL of 5% (*v*/*v*) formic acid. After that, the extraction of the peptides from the gel was carried out with a solution of 50% (*v*/*v*) acetonitrile/0.1% (*v*/*v*) trifluoroacetic acid (TFA) (×3) and acetonitrile (ACN) (×1). Samples were dried and stored at −20 °C until their further use [62].

### 4.7. Protein Quantification by SWATH-MS

SWATH/MS experiments were carried out following the instrumental parameters described elsewhere [13]. Briefly, two biological replicates of LA, LB-, LB+, HER+, TNBC and HC samples were used to get extensive quantitative data by label-free SWATH-MS analysis. Peptides of all samples were analyzed with a micro-LC system Ekspert nLC425 (Eksigen, Dublin, CA. USA) couplet to a hybrid quadrupole-TOF mass spectrometer Triple TOF 6600 (Sciex, Redwood City. CA. USA). One of the first steps is the construction of the MS/MS spectral libraries. For that purpose, peptide solutions were analyzed by a shotgun data-dependent acquisition (DDA) approach by micro-LC-MS/MS. For spectral alignment and peak extraction was employed the Peakview software (version 2.2; AB Sciex) using the SWATH Acquisition MicroApp (version 2.0). Parameters used were: number of fragments = 7, number of peptides = 10, peptide confidence = 95%, XIC width = 30 ppm and XIC extraction window = 5 min. Exportation of the SWATH file to the MarkerView software (version 1.3.1; AB Sciex) allowed the quantitative analysis of ions, peptides and proteins in the different samples. As output result, the summed intensity of ions for the peptide, summed intensity of the peptides for protein and Area under Curve (AUC) of the ions were provided. The test set (LA, LB-, LB+, HER+ and TNBC) was compared with the control (HC) dataset to generate fold change ratios. For protein quantitation, only peptides with a False Discovery Rate (FDR) below 1% were considered. Average MS peak area of each protein derived from the analysis of the biological replicates and Student’s *t*-test analysis among samples was developed. *t*-test indicates the capacity of each variable to distinguish between two groups, and it was reported as a *p*-value. The criterion to select differentially expressed proteins was a *p*-value <0.05 with a 1.5-fold in- or decrease.

### 4.8. Protein Functional Interaction Network Analysis and Protein Ontology Classification

The informatic tool STRING v.10.0 database (http://string-db.org) was the used to analyze the functional interaction networks of the proteins, integrating direct (physical) and indirect protein–protein interactions (PPI) [63].

Protein ontology classification was performed with the PANTHER classification system (http://www.pantherdb.org/). The differentially expressed proteins in the different breast cancer subtypes were grouped according to their major molecular function, biological processes, cellular origin, protein class and biological pathways.

## 5. Conclusions

The quantitative comparison of the ex vivo PCs formed upon incubation of AuNPs with serum samples obtained from BC patients revealed 75 deregulated subtype-specific unique proteins (8, 27, 2, 28 and 10 proteins specifically associated to the LA, LB-, LB+, HER2+ and TNBC subtypes, respectively). The analysis of the ex vivo PCs formed onto AuNPs revealed a profile of blood coagulation proteins in the serum of HER2-overexpressing BC patients that are implicated in breast tumor progression, including cellular transformation, proliferation, tumor cell survival and angiogenesis. Of all BC patients, HER2+ patients have the worst outcome. Further insights exploring these blood coagulation proteins as potential effective prognosis biomarkers and targets for novel therapeutic approaches could have a great impact on the management of HER2-overexpressing BC patients.

## Figures and Tables

**Table 1:** Number of differentially expressed proteins (up- and downregulated) (*p*-value ≤ 0.05) found in the protein patterns of the ex vivo formed coronas after the analysis by SWATH-MS for the different breast cancer subtypes (LA LB-, LB+, HER2+ and TNBC) in comparison with healthy controls (HC) samples. The number of differentially expressed proteins (up- and downregulated) specific to each of the five subtypes of BC found in the ex vivo formed coronas is also indicated.

SWATH-MS Analysis						
Comparison	Protein Number (*p*-Value ≤ 0.05)					
	Total	Upregulated	Downregulated	Specific	Upregulated	Downregulated
Controls vs. LA	60	42	18	8	4	4
Controls vs. LB-	132	100	32	27	25	2
Controls vs. LB+	67	59	8	2	2	0
Controls vs. HER2+	130	95	35	28	23	5
Controls vs. TNBC	91	87	4	10	9	1

**Table 2:** Differentially expressed proteins (up- and downregulated) (*p*-value ≤ 0.05) found in the protein patterns of the ex vivo formed coronas after the analysis by SWATH-MS specific (or unique) for the different breast cancer subtypes (LA, LB-, LB+, HER2+ and TNBC) in comparison with healthy control (HC) samples. The fold change ratio was calculated as the ratio of geometric means of the sample replicates, which corresponds to calculating the normal arithmetic ratio of log-transformed areas and back-transforming.

Protein Name	Gene	*p*-Value	Fold Change	
Complement C1r subcomponent-like protein	C1RL	0.0000979	1.614689351	↑ Luminal A
Complement factor H-related protein 2	CFHR2	0.003228805	1.764346734	↑ Luminal A
Complement component C8 beta chain	C8B	0.003730112	1.35440489	↑ Luminal A
Lysosome-associated membrane glycoprotein 2	LAMP2	0.018383379	1.33466653	↑ Luminal A
Immunoglobulin kappa variable 3–20	IGKV3–20	0.008581213	7.24153822	↓ Luminal A
Immunoglobulin heavy constant mu	IGHM	0.038320909	2.180105502	↓ Luminal A
Immunoglobulin heavy variable 1–24	IGHV1–24	0.045225189	2.766559939	↓ Luminal A
Protein Z-dependent protease inhibitor	SERPINA10	0.045960336	2.020513374	↓ Luminal A
Immunoglobulin lambda variable 2–23	IGLV2–23	0.000000251	2.96506068	↑ Luminal B HER2 Neg
Immunoglobulin heavy variable 3–53	IGHV3–53	0.00000690	3.231686892	↑ Luminal B HER2 Neg
Immunoglobulin kappa variable 4–1	IGKV4–1	0.00000788	2.271628981	↑ Luminal B HER2 Neg
Biotinidase	BTD	0.0000142	1.571319968	↑ Luminal B HER2 Neg
Immunoglobulin heavy constant alpha 1	IGHA1	0.0000181	2.45029046	↑ Luminal B HER2 Neg
Serum paraoxonase/lactonase 3	PON3	0.0000344	1.614088523	↑ Luminal B HER2 Neg
Immunoglobulin kappa constant	IGKC	0.0000604	2.234283878	↑ Luminal B HER2 Neg
Phospholipid transfer protein	PLTP	0.000127276	1.491697089	↑ Luminal B HER2 Neg
Immunoglobulin kappa variable 3–11	IGKV3–11	0.000235484	2.727555354	↑ Luminal B HER2 Neg
Immunoglobulin heavy variable 3–9	IGHV3–9	0.000418498	2.709287474	↑ Luminal B HER2 Neg
Alpha-mannosidase 2	MAN2A1	0.000577191	2.094925838	↑ Luminal B HER2 Neg
Immunoglobulin heavy constant gamma 1	IGHG1	0.000838666	1.832731289	↑ Luminal B HER2 Neg
Apolipoprotein B-100	APOB	0.001926757	1.618943103	↑ Luminal B HER2 Neg
Immunoglobulin heavy constant alpha 2	IGHA2	0.00207989	2.137794634	↑ Luminal B HER2 Neg
Basement membrane-specific heparan sulfate proteoglycan core protein	HSPG2	0.002185804	2.617271104	↑ Luminal B HER2 Neg
Pregnancy zone protein	PZP	0.002663618	4.343426266	↑ Luminal B HER2 Neg
Immunoglobulin heavy variable 1–69D	IGHV1–69D	0.00307313	2.762019962	↑ Luminal B HER2 Neg
Immunoglobulin heavy variable 3–74	IGHV3–74	0.005843078	1.63300774	↑ Luminal B HER2 Neg
Immunoglobulin lambda-like polypeptide 5	IGLL5	0.006474796	1.674164832	↑ Luminal B HER2 Neg
Immunoglobulin kappa variable 3D-20	IGKV3D-20	0.007387257	1.722873358	↑ Luminal B HER2 Neg
L-lactate dehydrogenase B chain	LDHB	0.011088233	1.464826821	↑ Luminal B HER2 Neg
Platelet glycoprotein Ib alpha chain	GP1BA	0.014355443	1.435497779	↑ Luminal B HER2 Neg
Apolipoprotein D	APOD	0.02689663	1.565894604	↑ Luminal B HER2 Neg
Immunoglobulin lambda variable 3–21	IGLV3–21	0.030887039	1.531598796	↑ Luminal B HER2 Neg
Mediator of RNA polymerase II transcription subunit 23	MED23	0.034189832	1.582094144	↑ Luminal B HER2 Neg
Platelet factor 4 variant	PF4V1	0.013149247	8.695958011	↓ Luminal B HER2 Neg
Complement C1s subcomponent	C1S	0.040782815	1.400513823	↓ Luminal B HER2 Neg
Plastin-2	LCP1	0.002018957	4.501473239	↑ Luminal B HER2 Pos
Immunoglobulin heavy variable 6–1	IGHV6–1	0.020191442	1.539338405	↑ Luminal B HER2 Pos
Complement C5	C5	0.00000000000000295	2.104078338	↑ HER2 Pos
Adiponectin	ADIPOQ	0.00000000828	9.579128591	↑ HER2 Pos
Immunoglobulin heavy variable 3–73	IGHV3–73	0.00000167	15.87006684	↑ HER2 Pos
Coagulation factor XII	F12	0.00000250	4.48323521	↑ HER2 Pos
Plasma kallikrein	KLKB1	0.0000205	2.839283512	↑ HER2 Pos
Immunoglobulin heavy variable 3–23	IGHV3–23	0.0000883	2.952483084	↑ HER2 Pos
Immunoglobulin lambda variable 1–51	IGLV1–51	0.000382645	2.419267666	↑ HER2 Pos
Immunoglobulin heavy variable 3–64	IGHV3–64	0.000401774	2.217759109	↑ HER2 Pos
Selenoprotein P	SELENOP	0.000539614	3.796988329	↑ HER2 Pos
Immunoglobulin kappa variable 1D-12	IGKV1D-12	0.00188481	4.471370936	↑ HER2 Pos
Immunoglobulin lambda variable 5–45	IGLV5–45	0.00772078	2.858034161	↑ HER2 Pos
Immunoglobulin lambda variable 6–57	IGLV6–57	0.009251968	5.252644969	↑ HER2 Pos
Keratin type I cytoskeletal 10	KRT10	0.012361191	1.599012598	↑ HER2 Pos
Immunoglobulin kappa variable 1–27	IGKV1–27	0.014636279	3.663302525	↑ HER2 Pos
Immunoglobulin kappa variable 1–5	IGKV1–5	0.015089737	3.097611266	↑ HER2 Pos
EGF-containing fibulin-like extracellular matrix protein 1	EFEMP1	0.015909032	1.962284999	↑ HER2 Pos
Immunoglobulin kappa variable 2–24	IGKV2–24	0.019946847	3.792528291	↑ HER2 Pos
Immunoglobulin heavy constant gamma 2	IGHG2	0.020013155	1.605775681	↑ HER2 Pos
Adipocyte plasma membrane-associated protein	APMAP	0.021204299	23.89017843	↑ HER2 Pos
Immunoglobulin kappa variable 1D-16	IGKV1D-16	0.023927557	15.36602613	↑ HER2 Pos
Coagulation factor V	F5	0.025887503	3.739380413	↑ HER2 Pos
Cysteine-rich secretory protein 3	CRISP3	0.034870563	3.338300324	↑ HER2 Pos
Immunoglobulin heavy variable 3–33	IGHV3–33	0.038394512	8.344444793	↑ HER2 Pos
N-acetylmuramoyl-L-alanine amidase	PGLYRP2	0.000321687	1.799194526	↓ HER2 Pos
Alpha-1-antitrypsin	SERPINA1	0.001111283	4.959761437	↓ HER2 Pos
Trypsin-1	PRSS1	0.002454431	4.217786063	↓ HER2 Pos
Apolipoprotein F	APOF	0.005336626	7.55893037	↓ HER2 Pos
Antithrombin-III	SERPINC1	0.018612975	1.308597079	↓ HER2 Pos
Apolipoprotein E	APOE	0.005108321	1.297484301	↑ Triple Negative
Voltage-dependent L-type calcium channel subunit alpha-1F	CACNA1F	0.010760078	3.330169664	↑ Triple Negative
Complement C2	C2	0.024115534	1.265184448	↑ Triple Negative
Keratin. type II cytoskeletal 1	KRT1	0.02492244	1.394646766	↑ Triple Negative
Immunoglobulin heavy variable 4–30-2	IGHV4–30-2	0.028590349	4.459516925	↑ Triple Negative
Attractin	ATRN	0.033317422	1.248304377	↑ Triple Negative
Immunoglobulin kappa variable 2D-30	IGKV2D-30	0.035772725	1.512873113	↑ Triple Negative
Immunoglobulin kappa variable 1–6	IGKV1–6	0.039260496	1.537751007	↑ Triple Negative
Platelet basic protein	PPBP	0.04971791	23.75806076	↑ Triple Negative
CD5 antigen-like	CD5L	0.012543111	1.999836008	↓ Triple Negative

**Table 3:**
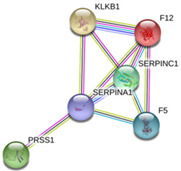
Candidate deregulated blood coagulation biomarkers in HER2-overexpressing BC patients found after the proteomic analysis of the ex vivo corona-coated AuNPs. On the bottom, a cluster of blood coagulation proteins found in the protein–protein interaction network map of the genes encoded differentially regulated proteins for the HER2-overexpressing BC patients found after the proteomic analysis of the ex vivo corona-coated AuNPs.

Protein Name	Gene	*p*-Value	Fold Change	Control vs. HER2 Positive
Coagulation factor XII	F12	0.00000250	4.48323521	↑ HER2 Pos
Plasma kallikrein	KLKB1	0.0000205	2.839283512	↑ HER2 Pos
Coagulation factor V	F5	0.025887503	3.739380413	↑ HER2 Pos
Alpha-1-antitrypsin	SERPINA1	0.001111283	4.959761437	↓ HER2 Pos
Trypsin-1	PRSS1	0.002454431	4.217786063	↓ HER2 Pos
Antithrombin-III	SERPINC1	0.018612975	1.308597079	↓ HER2 Pos

## Supplementary Materials

The following are available online at https://www.mdpi.com/1422-0067/21/22/8449/s1. Figure S1. Classification according to the molecular function of the differentially regulated proteins specific to each of the five subtypes of BC found in the ex vivo formed coronas analyzed with the PANTHER database, Figure S2. Classification according to the biological process of the differentially regulated proteins specific to each of the five subtypes of BC found in the ex vivo formed coronas analyzed with the PANTHER database, Figure S3. Classification according to the cellular component of the differentially regulated proteins specific to each of the five subtypes of BC found in the ex vivo formed coronas analyzed with the PANTHER database, Figure S4. Classification according to the biological pathway of the differentially regulated proteins specific to each of the five subtypes of BC found in the ex vivo formed coronas analyzed with the PANTHER database, Table S1. Clinical features of breast cancer tumors, Table S2. The average mean hydrodynamic diameter (nm) values of bare and protein corona-coated AuNPs, recovered post-incubation with human serum obtained from HC and BC patients, Table S3. Differentially expressed proteins (up- and downregulated) (*p*-value ≤ 0.05) found in the protein patterns of the ex vivo formed coronas after the analysis by SWATH-MS for the different breast cancer subtypes (LA, *n* = 11; LB-, *n* = 10; LB+, *n* = 7; HER2+, *n* = 6; TNBC, *n* = 8) in comparison with healthy control (HC) samples. The accession number, species (Human) and fold change values are also reported, Table S4. Differentially expressed proteins (up- and downregulated) (*p*-value ≤ 0.05) found in the protein patterns of the ex vivo formed coronas after the analysis by SWATH-MS for the different breast cancer subtypes (LA, *n* = 11; LB-, *n* = 10; LB+, *n* = 7; HER2+, *n* = 6; TNBC, *n* = 8) in comparison with healthy control (HC) samples, Table S5. Differentially expressed proteins (up- and downregulated) (*p*-value ≤ 0.05) found in the protein patterns of the ex vivo formed coronas after the analysis by SWATH-MS common and specific for the different breast cancer subtypes (LA, *n* = 11; LB-, *n* = 10; LB+, *n* = 7; HER2+, *n* = 6; TNBC, *n* = 8) in comparison with healthy control (HC) samples. The accession number, gene name and species (Human) are reported.

## Abbreviations

APPs	Acute-phase proteins
AuNPs	Gold nanoparticles
BC	Breast cancer
DTT	Dithiothreitol
ER	Estrogen receptor
HC	Healthy controls
HER2	Human epidermal growth factor receptor 2
IAA	Iodoacetic acid
LA	Luminal A
LB	Luminal B
MS	Mass spectrometry
PC	Protein corona
PR	Progesterone receptor
SDS-PAGE	Sodium dodecyl sulfate–polyacrylamide gel electrophoresis
SWATH-MS	Sequential Window Acquisition of All Theoretical Mass Spectra
TEM	Transmission electron microscopy
TNBC	Triple negative breast cancer
TF	Serotransferrin

## References

[1] Feng Y., Spezia M., Huang S., Yuan C., Zeng Z., Zhang L., Ji X., Liu W., Huang B., Luo W. (2018). Breast cancer development and progression: Risk factors, cancer stem cells, signaling pathways, genomics, and molecular pathogenesis. Genes Dis..

[2] Inic Z., Zegarac M., Inic M., Markovic I., Kozomara Z., Djurisic I., Inic I., Pupic G., Jancic S. (2014). Difference between Luminal A and Luminal B Subtypes According to Ki-67, Tumor Size, and Progesterone Receptor Negativity Providing Prognostic Information. Clin. Med. Insights Oncol..

[3] Colleoni M., Sun Z., Price K.N., Karlsson P., Forbes J.F., Thürlimann B., Gianni L., Castiglione M., Gelber R.D., Coates A.S. (2016). Annual Hazard Rates of Recurrence for Breast Cancer During 24 Years of Follow-Up: Results from the International Breast Cancer Study Group Trials I to V. J. Clin. Oncol..

[4] Loibl S., Gianni L. (2017). HER2-positive breast cancer. Lancet.

[5] Asif H., Sultana S., Ahmed S., Akhtar N., Tariq M. (2016). HER-2 Positive Breast Cance—A Mini-Review. Asian Pac. J. Cancer Prev..

[6] Wolff A.C., Hammond M.E.H., Allison K.H., Harvey B.E., Mangu P.B., Bartlett J.M.S., Bilous M., Ellis I.O., Fitzgibbons P., Hanna W. (2018). Human epidermal growth factor receptor 2 testing in breast cancer: American society of clinical oncology/college of american pathologists clinical practice guideline focused update. J. Clin. Oncol..

[7] Suman S., Basak T., Gupta P., Mishra S., Kumar V., Sengupta S., Shukla Y. (2016). Quantitative Proteomics revealed novel proteins associated with molecular subtypes of breast cancer. J. Proteom..

[8] Gajbhiye A., Dabhi R., Taunk K., Jagadeeshaprasad M.G., RoyChoudhury S., Mane A., Bayatigeri S., Chaudhury K., Santra M.K., Rapole S. (2017). Multipronged quantitative proteomics reveals serum proteome alterations in breast cancer intrinsic subtypes. J. Proteom..

[9] Bouchal P., Schubert O.T., Faktor J., Capkova L., Imrichova H., Zoufalova K., Paralova V., Hrstka R., Liu Y., Ebhardt H.A. (2019). Breast Cancer Classification Based on Proteotypes Obtained by SWATH Mass Spectrometry. Cell Rep..

[10] Jia L., Lu Y., Shao J., Liang X.J., Xu Y. (2013). Nanoproteomics: A new sprout from emerging links between nanotechnology and proteomics. Trends Biotechnol..

[11] Pozzi D., Caracciolo G., Digiacomo L., Colapicchioni V., Palchetti S., Capriotti A.L., Cavaliere C., Zenezini Chiozzi R., Puglisi A., Laganà A. (2015). The biomolecular corona of nanoparticles in circulating biological media. Nanoscale.

[12] Chantada-Vázquez M.P., Castro López A., Bravo S.B., Vázquez-Estévez S., Acea-Nebril B., Núñez C. (2019). Proteomic analysis of the bio-corona formed on the surface of (Au, Ag, Pt)-nanoparticles in human serum. Colloids Surf. B Biointerfaces.

[13] Chantada-Vázquez M.P., Castro López A., García Vence M., Vázquez-Estévez S., Acea-Nebril B., Calatayud D.G., Jardiel T., Bravo S.B., Núñez C. (2020). Proteomic investigation on bio-corona of Au, Ag and Fe nanoparticles for the discovery of triple negative breast cancer serum protein biomarkers. J. Proteom..

[14] Chantada-Vázquez M.P., García-Vence M., Vázquez-Estévez S., Bravo S.B., Núñez C. (2020). Identification of a Profile of Neutrophil-Derived Granule Proteins in the Surface of Gold Nanoparticles after Their Interaction with Human Breast Cancer Sera. Nanomaterials (Basel).

[15] Docter D., Westmeier D., Markiewicz M., Stolte S., Knauer S.K., Stauber R.H. (2015). The nanoparticle biomolecule corona: Lessons learned–challenge accepted?. Chem. Soc. Rev..

[16] Lai Z.W., Yan Y., Caruso F., Nice E.C. (2012). Emerging techniques in proteomics for probing nano-bio interactions. ACS Nano.

[17] Bai X., Wang Y., Song Z., Feng Y., Chen Y., Zhang D., Feng L. (2020). The Basic Properties of Gold Nanoparticles and their Applications in Tumor Diagnosis and Treatment. Int. J. Mol. Sci..

[18] Dobrovolskaia M.A., Patri A.K., Zheng J., Clogston J.D., Ayub N., Aggarwal P., Neun B.W., Hall J.B., McNeil S.E. (2009). Interaction of colloidal gold nanoparticles with human blood: Effects on particle size and analysis of plasma protein binding profiles. Nanomedicine.

[19] García-Álvarez R., Hadjidemetriou M., Sánchez-Iglesias A., Liz-Marzán L.M., Kostarelos K. (2018). In vivo formation of protein corona on gold nanoparticles. The effect of size and shape. Nanoscale.

[20] Mi H., Muruganujan A., Thomas P.D. (2013). PANTHER in 2013: Modeling the evolution of gene function, and other gene attributes, in the context of phylogenetic trees. Nucleic Acids Res..

[21] Coussens L.M., Raymond W.W., Bergers G. (1999). Inflammatory mast cells up-regulate angiogenesis during squamous epithelial carcinogenesis. Genes Dev..

[22] Singh B., Berry J.A., Shoher A., Lucci A. (2006). COX-2 induces IL-11 production in human breast cancer cells. J. Surg. Res..

[23] Singh R.P., Singh V.P., Udupa K.N. (1991). E-Rosette forming lymphocytes and serum immunoglobulins in breast cancer patients. Mater. Med. Pol..

[24] Lu Y., Hu X. (2014). C5a stimulates the proliferation of breast cancer cells via Akt-dependent RGC-32 gene activation. Oncol. Rep..

[25] Nasim F., Ejaz S., Ashraf M., Asif A.R., Oellerich M., Ahmad G., Malik G.A., Rehman A.-U. (2012). Potential Biomarkers in the Sera of Breast Cancer Patients from Bahawalpur, Pakistan. Biomark. Cancer.

[26] Liu A., Qu H., Yu C., Liu J., Jiao A., Sun P. (2017). Serum biomarkers for lymph node metastasis in patients with triple-negative breast cancer by proteomics. Int. J. Clin. Exp. Pathol..

[27] Tiedemann K., Sadvakassova G., Mikolajewicz N., Juhas M., Sabirova Z., Tabariès S., Gettemans J., Siegel P.M., Komarova S.V. (2019). Exosomal Release of L-Plastin by Breast Cancer Cells Facilitates Metastatic Bone Osteolysis. Transl. Oncol..

[28] Ishikawa S., Miyashita T., Inokuchi M., Hayashi H., Oyama K., Tajima H., Takamura H., Ninomiya I., Ahmed A.K., Harman J.W. (2016). Platelets surrounding primary tumor cells are related to chemoresistance. Oncol. Rep..

[29] Goodarzi A., Yari F., Mohammadipour M., Deyhim M.R., Naghadeh H.T. (2018). Capability of Platelet Factor 4 to Induce Apoptosis in the Cancerous Cell Lines in Vitro. Int. J. Med. Lab..

[30] Pendharkar N., Gajbhiye A., Taunk K., RoyChoudhury S., Dhali S., Seal S., Mane A., Abhang S., Santra M.K., Chaudhury K. (2016). Quantitative tissue proteomic investigation of invasive ductal carcinoma of breast with luminal B HER2 positive and HER2 enriched subtypes towards potential diagnostic and therapeutic biomarkers. J. Proteome.

[31] Zhang L., Lu H., Yang P. (2010). Recent developments of nanoparticle-based enrichment methods for mass spectrometric analysis in proteomic. Sci. China Chem..

[32] Asegaonkar S.B., Takalkar U.V., Kodlikeri P., Pagdhune A., Bonduliya V., Thorat A.P. (2014). Serum high sensitivity C-reactive protein in breast cancer patients. Int. J. Res. Med. Sci..

[33] Kaur R.P., Rubal, Banipal R.P.S., Vashistha R., Dhiman M., Munshi A. (2019). Association of elevated levels of C-reactive protein with breast cancer, breast cancer subtypes and poor outcome. Curr. Probl. Cancer.

[34] Zhang G., Sun X., Lv H., Yang X., Kang X. (2012). Serum amyloid A: A new potential serum marker correlated with the stage of breast cancer. Oncol. Lett..

[35] Ponzetti M., Capulli M., Angelucci A., Ventura L., Monache S.D., Mercurio C., Calgani A., Sanita P., Teti A., Rucci N. (2017). Non-conventional role of haemoglobin beta in breast malignancy. Br. J. Cancer.

[36] Vandewalle B., Hornez L., Revillion F., Lefebvre J. (1989). Secretion of transferrin by human breast cancer cells. Biochem. Biophys. Res. Commun..

[37] Lima L.G., Monteiro R.Q. (2013). Activation of blood coagulation in cancer: Implications for tumour progression. Biosci. Rep..

[38] Metelli A., Wu B.X., Riesenberg B., Guglietta S., Huck J.D., Mills C., Li A., Rachidi S., Krieg C., Rubinstein M.P. (2020). Thrombin contributes to cancer immune evasion via proteolysis of platelet-bound GARP to activate LTGF-β. Sci. Transl. Med..

[39] Hisada Y., Mackman N. (2017). Cancer-associated pathways and biomarkers of venous thrombosis. Blood.

[40] Ruf W., Rothmeier A.S., Graf C. (2016). Targeting clotting proteins in cancer therapy—Progress and challenges. Thromb. Res..

[41] Lal I., Dittus K., Holmes C.E. (2013). Platelets, coagulation and fibrinolysis in breast cancer progression. Breast Cancer Res..

[42] Tas F., Kilic L., Duranyildiz D. (2014). Coagulation tests show significant differences in patients with breast cancer. Tumor Biol..

[43] Liu Y.-L., Lu Q., Liang J.-W., Xia Y., Zhang W., Hu B.-Q., Shang F.-F., Ji Y.-R., Wang J., Wang Q. (2015). High Plasma Fibrinogen is Correlated with Poor Response to Trastuzumab Treatment in HER2 Positive Breast Cancer. Medicine (Baltimore).

[44] Yigit E., Gönüllü G., Yücel I., Turgut M., Erdem D., Cakar B. (2008). Relation between hemostatic parameters and prognostic/predictive factors in breast cancer. Eur. J. Intern. Med..

[45] Tinholt M., Garred Ø., Borgen E., Beraki E., Sletten M., Kleivi Sahlberg K., Sandset P.M., Iversen N. (2011). Coagulation factor V is expressed in tumors and predicts favorable outcome in aggressive breast cancer. Thromb. Res..

[46] Mutch N.J. (2011). Emerging roles for factor XII in vivo. J. Thromb. Haemost..

[47] Kryza T., Silva M.L., Loessner D., Heuzé-Vourc’h N., Clements J.A. (2016). The kallikrein-related peptidase family: Dysregulation and functions during cancer progression. Biochimie.

[48] Zelvyte I., Sjögren H.O., Janciauskiene S. (2002). Effects of native and cleaved forms of alpha1-antitrypsin on ME 1477 tumor cell functional activity. Cancer Detect. Prev..

[49] Chan H.J., Li H., Liu Z., Yuan Y.C., Mortimer J., Chen S. (2015). SERPINA1 is a direct estrogen receptor target gene and a predictor of survival in breast cancer patients. Oncotarget.

[50] Luengo-Gil G., Calvo M.I., Martín-Villar E., Águila S., Bohdan N., Antón A.I., Espín S., de la Peña F.A., Vicente V., Corral J. (2016). Antithrombin controls tumor migration, invasion and angiogenesis by inhibition of enteropeptidase. Sci. Rep..

[51] Gu L., Cao C., Fu J., Li Q., Li D.-H., Chen M.-Y. (2018). Serum adiponectin in breast cancer. A meta-analysis. Medicine (Baltimore).

[52] Oh S.W., Park C.-Y., Lee E.S., Yoon Y.S., Lee E.S., Park S.S., Kim Y., Sung N.J., Yun Y.H., Lee K.S. (2011). Adipokines, insulin resistance, metabolic syndrome, and breast cancer recurrence: A cohort study. Breast Cancer Res..

[53] Zhou Y., Luo G. (2020). Apolipoproteins, as the carrier proteins for lipids, are involved in the development of breast cancer. Clin. Transl. Oncol..

[54] Cibeira G.H., Giacomazzi J., Aguiar E., Schneider S., Ettrich B., De Souza C.I., Camey S., Caleffi M., Weber B., Ashton-Prolla P. (2014). Apolipoprotein E genetic polymorphism, serum lipoprotein levels and breast cancer risk: A case-control study. Mol. Clin. Oncol..

[55] Liu J.-X., Yuan Q., Min Y.-L., He Y., Xu Q.-H., Li B., Shi W.-Q., Lin Q., Li Q.-H., Zhu P.-W. (2019). Apolipoprotein A1 and B as risk factors for development of intraocular metastasis in patients with breast cancer. Cancer Manag. Res..

[56] Saha T. (2012). LAMP2A overexpression in breast tumors promotes cancer cell survival via chaperone-mediated autophagy. Autophagy.

[57] Whitelock J.M., Graham L.D., Melrose J., Murdoch A.D., Iozzo R.V., Underwood P.A. (1999). Human perlecan immunopurified from different endothelial cell sources has different adhesive properties for vascular cells. Matrix Biol..

[58] Kalscheuer S., Khanna V., Kim H., Li S., Sachdev D., DeCarlo A., Yang D., Panyam J. (2019). Discovery of HSPG2 (Perlecan) as a Therapeutic Target in Triple Negative Breast Cancer. Sci. Rep..

[59] Davidson B., Stavnes H.T., Holth A., Chen X., Yang Y., Shih I.-M., Wang T.-L. (2011). Gene expression signatures differentiate ovarian/peritoneal serous carcinoma from breast carcinoma in effusions. J. Cell. Mol. Med..

[60] Aran G., Sanjurjo L., Bárcena C., Simon-Coma M., Téllez E., Vázquez-Vitali M., Garrido M., Guerra L., Díaz E., Ojanguren I. (2018). CD5L is upregulated in hepatocellular carcinoma and promotes liver cancer cell proliferation and antiapoptotic responses by binding to HSPA5 (GRP78). FASEB J..

[61] Dennison J.B., Molina J.R., Mitra S., González-Angulo A.M., Balko J.M., Kuba M.G., Sanders M.E., Pinto J.A., Gómez H.L., Arteaga C.L. (2013). Lactate Dehydrogenase B: A Metabolic Marker of Response to Neoadjuvant Chemotherapy in Breast Cancer. Clin. Cancer Res..

[62] Oliveira E., Araújo J.E., Gómez-Meire S., Lodeiro C., Pérez-Melón C., Iglesias-Lamas E., Otero-Glez A., Capelo J.L., Santos H.M. (2014). Proteomics analysis of the peritoneal dialysate effluent reveals the presence of calcium-regulation proteins and acute inflammatory response. Clin. Proteom..

[63] Szklarczyk D., Franceschini A., Wyder S., Forslund K., Heller D., Huerta-Cepas J., Simonovic M., Roth A., Santos A., Tsafou K.P. (2015). STRING v10: Protein-protein interaction networks, integrated over the tree of life. Nucleic Acids Res..

