# Supplementary material for: Protein Corona Gold Nanoparticles Fingerprinting Reveals a Profile of Blood Coagulation Proteins in the Serum of HER2-Overexpressing Breast Cancer Patients
Source: Int J Mol Sci. 2020 Nov 10;21(22):8449. doi: 10.3390/ijms21228449 (PMC7696934; doi:10.3390/ijms21228449)
Supplement: Supplementary file 1 [file ijms-21-08449-s001.pdf]

Supplementary Information

Protein corona-coated gold nanoparticles fingerprinting reveals a profile of blood coagulation proteins in the serum of HER2-overexpressing breast cancer patients

María del Pilar Chantada-Vázquez,[a,b] Antonio Castro López,[c] María García-Vence,[b] Benigno Acea-Nebril,[d] Susana B. Bravo,[b*] Cristina Núñez[a*]

[a] Research Unit, Hospital Universitario Lucus Augusti (HULA), Servizo Galego de Saúde (SERGAS), 27002 Lugo, Spain

[b] Proteomic Unit, Instituto de Investigaciones Sanitarias-IDIS, Complejo Hospitalario Universitario de Santiago de Compostela (CHUS), 15706 Santiago de Compostela, Spain

[c] Breast Unit, Hospital Universitario Lucus Augusti (HULA), Servizo Galego de Saúde (SERGAS), 27002, Lugo, Spain

[d] Department of Surgery, Breast Unit, Complexo Hospitalario Universitario A Coruña (CHUAC), SERGAS, A Coruña, Spain

Keywords: Protein corona (PC); gold nanoparticles (AuNPs); breast cancer (BC); fingerprinting; SWATH-MS; HER2+.

Table of Contents

Figure S1. Classification according to the **molecular function** of the differentially regulated proteins specific to each of the five subtypes of BC found in the *ex vivo* formed coronas analyzed with the PANTHER database.

Figure S2. Classification according to the **biological process** of the differentially regulated proteins specific to each of the five subtypes of BC found in the *ex vivo* formed coronas analyzed with the PANTHER database

Figure S3. Classification according to the **cellular component** of the differentially regulated proteins specific to each of the five subtypes of BC found in the *ex vivo* formed coronas analyzed with the PANTHER database.

Figure S4. Classification according to the **biological pathway** of the differentially regulated proteins specific to each of the five subtypes of BC found in the *ex vivo* formed coronas analyzed with the PANTHER database.

Figure S1. Classification according to the **molecular function** of the differentially regulated proteins specific to each of the five subtypes of BC found in the *ex vivo* formed coronas analyzed with the PANTHER database.

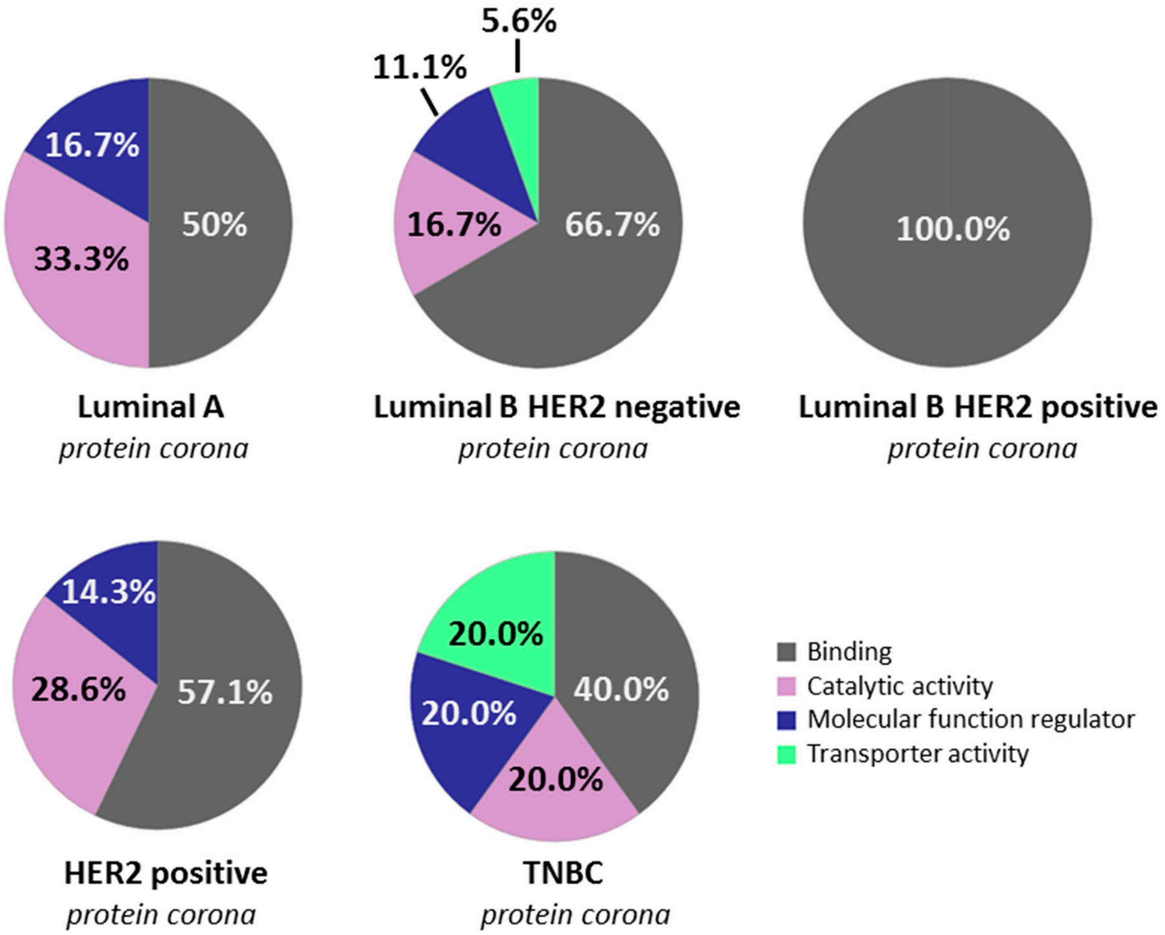

Figure S2. Classification according to the **biological process** of the differentially regulated proteins specific to each of the five subtypes of BC found in the *ex vivo* formed coronas analyzed with the PANTHER database.

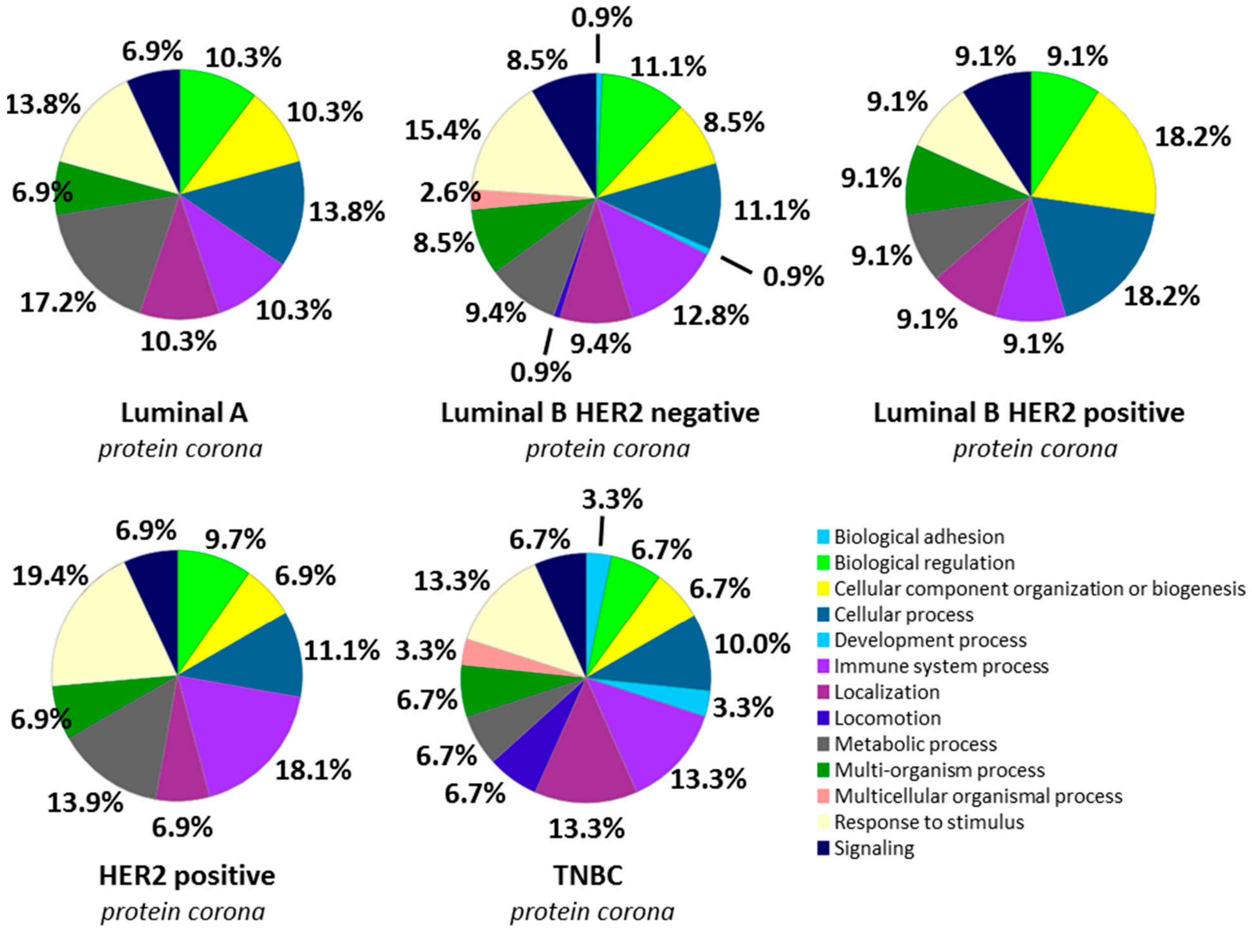

Figure S3. Classification according to the **cellular component** of the differentially regulated proteins specific to each of the five subtypes of BC found in the *ex vivo* formed coronas analyzed with the PANTHER database.

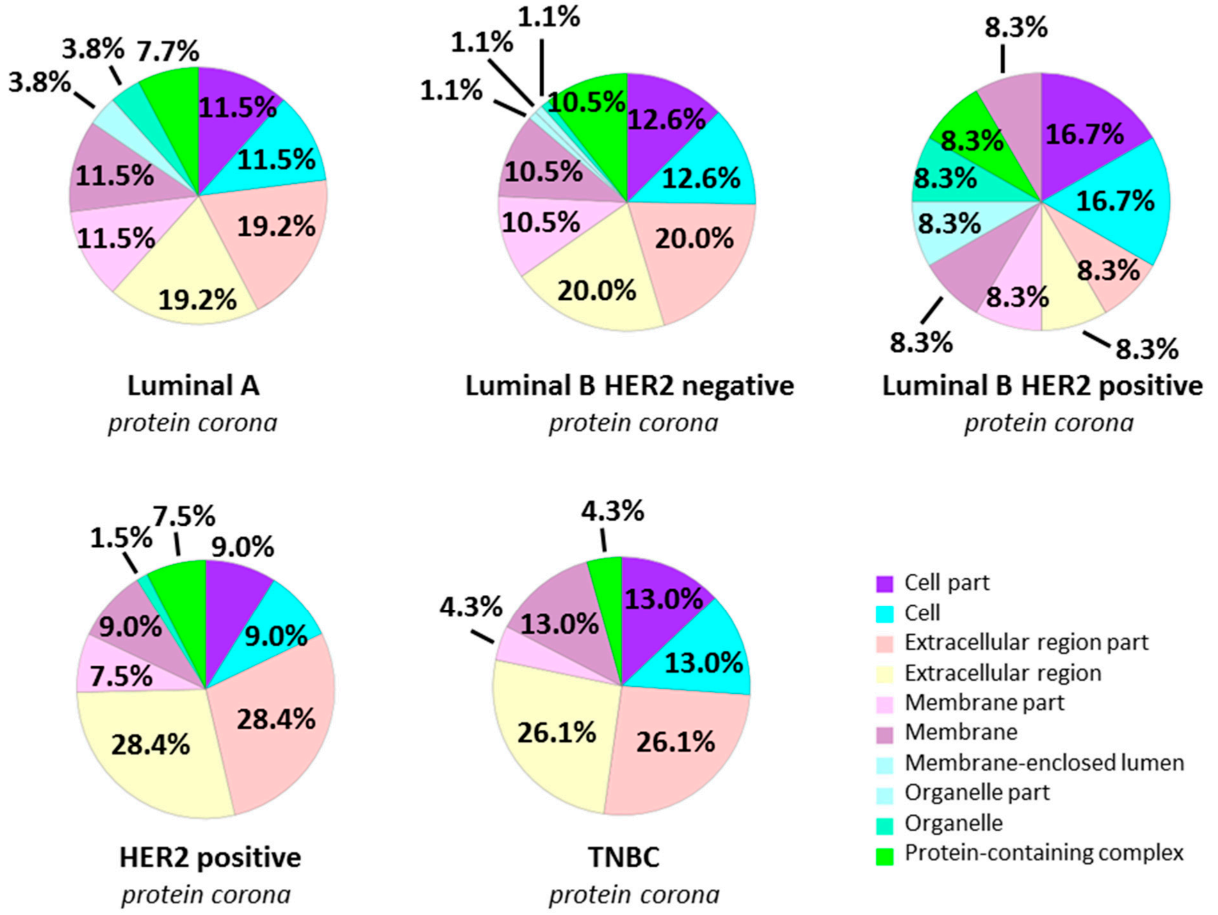

Figure S4. Classification according to the **biological pathway** of the differentially regulated proteins specific to each of the five subtypes of BC found in the *ex vivo* formed coronas analyzed with the PANTHER database.

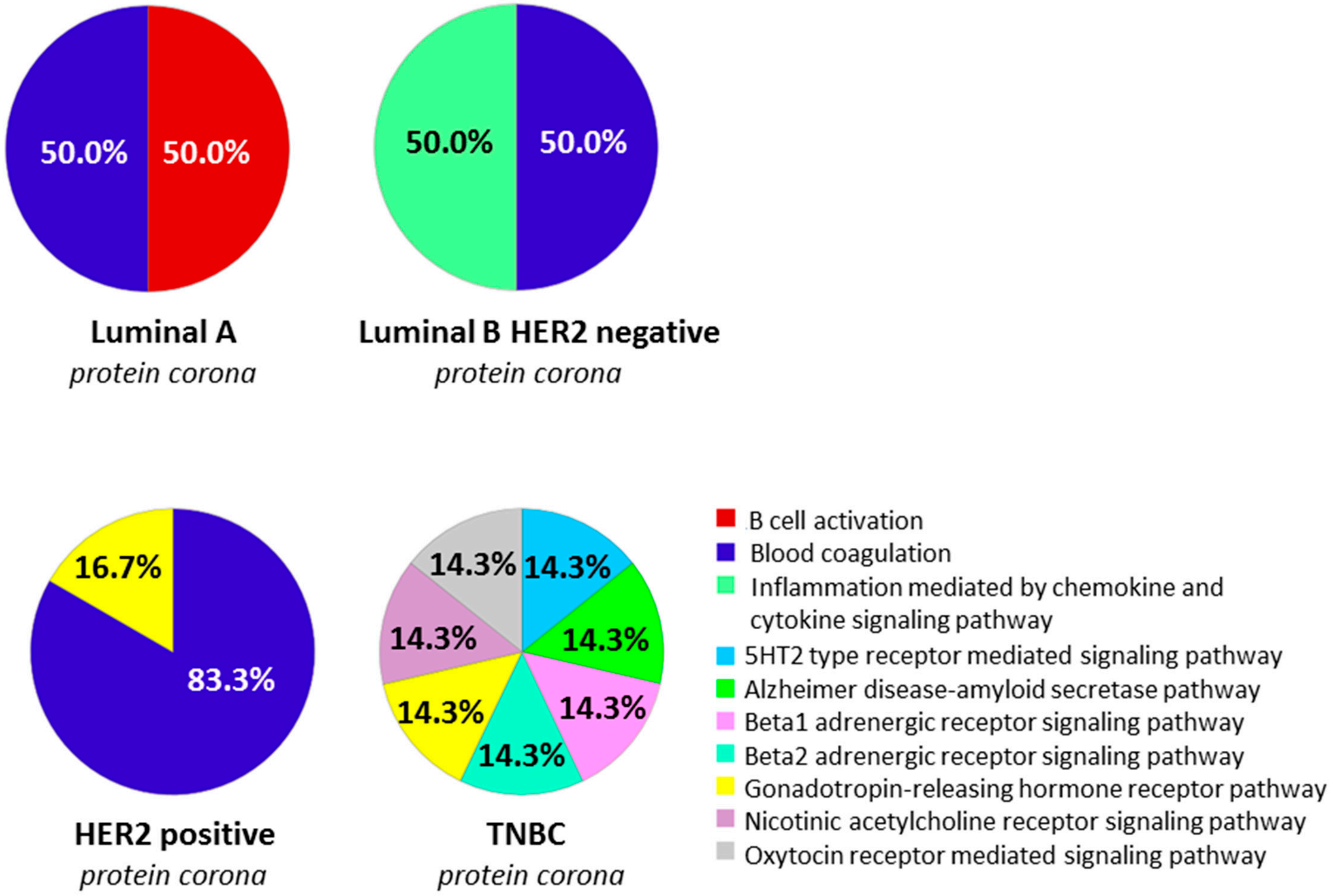

Table S1. Clinical features of breast cancer tumors.

Table S2. Table shows the average mean hydrodynamic diameter (nm) values of bare and protein corona-coated AuNPs, recovered post-incubation with human serum obtained from HC and BC patients.

Table S3. Differentially expressed proteins (up-regulated and down-regulated) (p-value ≤ 0.05) found in the protein patterns of the *ex vivo* formed coronas after the analysis by SWATH-MS for the different breast cancer subtypes (LA, n = 11; LB-, n = 10; LB+, n = 7; HER2+, n = 6; TNBC, n = 8) in comparison with healthy control (HC) samples. The accession number, species (Human) and fold change values were also reported.

Table S4. Differentially expressed proteins (up-regulated and down-regulated) (p-value ≤ 0.05) found in the protein patterns of the *ex vivo* formed coronas after the analysis by SWATH-MS for the different breast cancer subtypes (LA, n = 11; LB-, n = 10; LB+, n = 7; HER2+, n = 6; TNBC, n = 8) in comparison with healthy control (HC) samples.

Table S5. Differentially expressed proteins (up-regulated and down-regulated) (p-value ≤ 0.05) found in the protein patterns of the *ex vivo* formed coronas after the analysis by SWATH-MS common and specific for the different breast cancer subtypes (LA, n = 11; LB-, n = 10; LB+, n = 7; HER2+, n = 6; TNBC, n = 8) in comparison with healthy control (HC) samples. The accession number, gene name and species (Human) were reported.

Table S1. Clinical features of breast cancer tumors.

**Characteristics**		**Number**
Patients		
Age (years)	< 40	4
	40-59	21
	60-80	16
	> 80	1
Tumor size (cm)	< 2	25
	2-5	14
	>5	3
Histological types	*In situ* ductal carcinoma	2
	Invasive ductal carcinoma	36
	In situ lobular carcinoma	1
	Invasive lobular carcinoma	3
Receptor status	Luminal A	11
	Luminal B HER2 negative	10
	Luminal B HER2 positive	7
	HER2 positive	6
	Triple negative	8
Clinical stage	I	15
	II	20
	III	7
Nodal status	N0	25
	N1	17

Table S2. Table shows the average mean hydrodynamic diameter (nm) determined by dynamic light scaterring (DLS) of bare and protein corona-coated AuNPs, recovered post-incubation with human serum obtained from HC and BC patients.

Sample Name	Hydrodynamic Diameter (nm)
bare AuNPs	12.6
	13.8
	12.5
	**Mean ± s.d = 12.96 ± 0.72**
PC-coated AuNPs (HC)	17.8
	15.6
	18.6
	**Mean ± s.d = 17.33 ± 1.55**
PC-coated AuNPs (BC)	18.9
	16.3
	16.2
	**Mean ± s.d = 17.13 ± 1.53**

Table S3. Differentially expressed proteins (up-regulated and down-regulated) (p-value ≤ 0.05) found in the protein patterns of the *ex vivo* formed coronas after the analysis by SWATH-MS for the different breast cancer subtypes (LA, n = 11; LB-, n = 10; LB+, n = 7; HER2+, n = 6; TNBC, n = 8) in comparison with healthy control (HC) samples. The accession number, species (Human) and fold change values were also reported. Only proteins with $p \leq 0.05$ are shown.

	Protein Name	Entry Name	UniProt Name	*p*-value	Fold Change	
**Control *vs.* Luminal A**	Dopamine beta-hydroxylase	P09172	DOPO_HUMAN	1.18E-11	1.950147057	↑ Luminal A
	Fibrinogen alpha chain	P02671	FIBA_HUMAN	3.02E-08	2.045923215	↑ Luminal A
	C4b-binding protein alpha chain	P04003	C4BPA_HUMAN	6.57E-07	2.006126542	↑ Luminal A
	Complement component C9	P02748	CO9_HUMAN	1.09E-06	1.627212883	↑ Luminal A
	Plasma protease C1 inhibitor	P05155	IC1_HUMAN	2.31E-06	1.569699351	↑ Luminal A
	Cathelicidin antimicrobial peptide	P49913	CAMP_HUMAN	8.81E-06	1.588534625	↑ Luminal A
	Ficolin-2	Q15485	FCN2_HUMAN	1.60E-05	1.866106539	↑ Luminal A
	Complement C1r subcomponent-like protein	Q9NZP8	C1RL_HUMAN	9.79E-05	1.614689351	↑ Luminal A
	Plasminogen	P00747	PLMN_HUMAN	0.000145031	1.388556273	↑ Luminal A
	Nuclear receptor coactivator 6	Q14686	NCOA6_HUMAN	0.000155599	1.502869606	↑ Luminal A
	IgGFc-binding protein	Q9Y6R7	FCGBP_HUMAN	0.000452171	1.343798096	↑ Luminal A
	Beta-2-glycoprotein 1	P02749	APOH_HUMAN	0.000738185	1.48991011	↑ Luminal A
	Ficolin-3	O75636	FCN3_HUMAN	0.000776505	1.41771829	↑ Luminal A
	Apolipoprotein C-III	P02656	APOC3_HUMAN	0.000794669	1.702941426	↑ Luminal A
	Coagulation factor X	P00742	FA10_HUMAN	0.000869295	1.500969977	↑ Luminal A
	Complement C1r subcomponent	P00736	C1R_HUMAN	0.000915575	1.419961586	↑ Luminal A
	Hemopexin	P02790	HEMO_HUMAN	0.000948646	1.201990625	↑ Luminal A
	Vitamin K-dependent protein C	P04070	PROC_HUMAN	0.001634691	1.251503273	↑ Luminal A
	Haptoglobin	P00738	HPT_HUMAN	0.001967281	1.712283929	↑ Luminal A
	C-reactive protein	P02741	CRP_HUMAN	0.002021267	3.793350802	↑ Luminal A

	Complement factor B	P00751	CFAB_HUMAN	0.003001733	1.404455044	↑ Luminal A
	Complement factor H-related protein 2	P36980	FHR2_HUMAN	0.003228805	1.764346734	↑ Luminal A
	Complement component C8 beta chain	P07358	CO8B_HUMAN	0.003730112	1.35440489	↑ Luminal A
	Apolipoprotein L1	O14791	APOL1_HUMAN	0.004924638	1.379020279	↑ Luminal A
	Plasma serine protease inhibitor	P05154	IPSP_HUMAN	0.005373866	1.461857956	↑ Luminal A
	Inter-alpha-trypsin inhibitor heavy chain H3	Q06033	ITIH3_HUMAN	0.006148372	1.20110357	↑ Luminal A
	Hyaluronan-binding protein 2	Q14520	HABP2_HUMAN	0.006476169	1.356977288	↑ Luminal A
	Vitronectin	P04004	VTNC_HUMAN	0.006605812	1.33651024	↑ Luminal A
	Collectin-11	Q9BWP8	COL11_HUMAN	0.00792774	1.745818384	↑ Luminal A
	Serum amyloid P-component	P02743	SAMP_HUMAN	0.007991583	2.368589888	↑ Luminal A
	Histidine-rich glycoprotein	P04196	HRG_HUMAN	0.00943722	1.435955402	↑ Luminal A
	Alpha-2-antiplasmin	P08697	A2AP_HUMAN	0.010470312	1.30176602	↑ Luminal A
	Coagulation factor IX	P00740	FA9_HUMAN	0.011045501	1.456336295	↑ Luminal A
	Peroxiredoxin-2	P32119	PRDX2_HUMAN	0.013776963	1.415849095	↑ Luminal A
	Complement component C8 gamma chain	P07360	CO8G_HUMAN	0.016920167	1.322470801	↑ Luminal A
	Fibronectin	P02751	FINC_HUMAN	0.018192086	1.366200008	↑ Luminal A
	Lysosome-associated membrane glycoprotein 2	P13473	LAMP2_HUMAN	0.018383379	1.33466653	↑ Luminal A
	Vitamin K-dependent protein S	P07225	PROS_HUMAN	0.027466364	1.205727193	↑ Luminal A
	Hemoglobin subunit beta	P68871	HBB_HUMAN	0.031563001	1.41675834	↑ Luminal A
	Serotransferrin	P02787	TRFE_HUMAN	0.032339274	1.298146303	↑ Luminal A
	Serum amyloid A-4 protein	P35542	SAA4_HUMAN	0.034951962	1.464799784	↑ Luminal A
	Lipopolysaccharide-binding protein	P18428	LBP_HUMAN	0.036796813	1.341308401	↑ Luminal A
	Alpha-2-macroglobulin	P01023	A2MG_HUMAN	0.000421478	2.059179008	↑ Control
	Keratin. type I cytoskeletal 14	P02533	K1C14_HUMAN	0.001165874	1.925697694	↑ Control
	Sex hormone-binding globulin	P04278	SHBG_HUMAN	0.001691964	1.37783039	↑ Control
	Immunoglobulin heavy variable 7-4-1	A0A0J9YVY3	HV741_HUMAN	0.002730217	2.943962624	↑ Control
	Immunoglobulin heavy variable 4-28	A0A0C4DH34	HV428_HUMAN	0.003319191	2.434694876	↑ Control

	Immunoglobulin kappa variable 3-20	P01619	KV320_HUMAN	0.008581213	7.24153822	↑ Control
	Immunoglobulin heavy variable 3-49	A0A0A0MS15	HV349_HUMAN	0.014026719	1.743286666	↑ Control
	Immunoglobulin kappa variable 1-8	A0A0C4DH67	KV108_HUMAN	0.022022978	2.189831126	↑ Control
	Cadherin-5	P33151	CADH5_HUMAN	0.025861321	1.661020794	↑ Control
	Carboxypeptidase N catalytic chain	P15169	CBPN_HUMAN	0.02720155	1.197835464	↑ Control
	Beta-Ala-His dipeptidase	Q96KN2	CNDP1_HUMAN	0.030943391	1.505285886	↑ Control
	Immunoglobulin heavy variable 3-72	A0A0B4J1Y9	HV372_HUMAN	0.032233962	1.515212348	↑ Control
	Ceruloplasmin	P00450	CERU_HUMAN	0.035237156	1.836741181	↑ Control
	Immunoglobulin heavy constant mu	P01871	IGHM_HUMAN	0.038320909	2.180105502	↑ Control
	Carboxypeptidase N subunit 2	P22792	CPN2_HUMAN	0.04141732	1.267661976	↑ Control
	Corticosteroid-binding globulin	P08185	CBG_HUMAN	0.044573382	2.390576271	↑ Control
	Immunoglobulin heavy variable 1-24	A0A0C4DH33	HV124_HUMAN	0.045225189	2.766559939	↑ Control
	Protein Z-dependent protease inhibitor	Q9UK55	ZPI_HUMAN	0.045960336	2.020513374	↑ Control
**Control *vs.* Luminal B HER2 Negative**	**Protein Name**	**Entry Name**	**UniProt Name**	***p*-value**	**Fold Change**	
	Retinol-binding protein 4	P02753	RET4_HUMAN	2.36E-11	1.973091607	↑ Luminal B HER2 Neg
	Apolipoprotein L1	O14791	APOL1_HUMAN	2.92E-10	2.185971827	↑ Luminal B HER2 Neg
	Apolipoprotein A-II	P02652	APOA2_HUMAN	1.47E-08	1.906377953	↑ Luminal B HER2 Neg
	Immunoglobulin lambda variable 2-23	P01705	LV223_HUMAN	2.51E-07	2.96506068	↑ Luminal B HER2 Neg
	Angiotensinogen	P01019	ANGT_HUMAN	3.00E-07	2.34185762	↑ Luminal B HER2 Neg
	Immunoglobulin lambda variable 1-47	P01700	LV147_HUMAN	3.57E-07	3.087251144	↑ Luminal B HER2 Neg
	Immunoglobulin lambda variable 3-10	A0A075B6K4	LV310_HUMAN	5.46E-07	3.944815387	↑ Luminal B HER2 Neg
	Inter-alpha-trypsin inhibitor heavy chain H1	P19827	ITIH1_HUMAN	1.19E-06	1.589603796	↑ Luminal B HER2 Neg
	Cathelicidin antimicrobial peptide	P49913	CAMP_HUMAN	1.22E-06	1.910900014	↑ Luminal B HER2 Neg
	Inter-alpha-trypsin inhibitor heavy chain H2	P19823	ITIH2_HUMAN	1.41E-06	1.580896901	↑ Luminal B HER2 Neg
	Apolipoprotein C-II	P02655	APOC2_HUMAN	1.43E-06	1.785136638	↑ Luminal B HER2 Neg
	Serum amyloid A-4 protein	P35542	SAA4_HUMAN	2.91E-06	2.245805522	↑ Luminal B HER2 Neg
	Alpha-1B-glycoprotein	P04217	A1BG_HUMAN	3.09E-06	2.508360284	↑ Luminal B HER2 Neg

	Immunoglobulin lambda constant 7	A0M8Q6	IGLC7_HUMAN	3.52E-06	3.764178124	↑ Luminal B HER2 Neg
	IgGFc-binding protein	Q9Y6R7	FCGBP_HUMAN	4.86E-06	1.502574907	↑ Luminal B HER2 Neg
	Complement component C6	P13671	CO6_HUMAN	6.19E-06	3.744383593	↑ Luminal B HER2 Neg
	Immunoglobulin heavy variable 3-53	P01767	HV353_HUMAN	6.90E-06	3.231686892	↑ Luminal B HER2 Neg
	Immunoglobulin kappa variable 4-1	P06312	KV401_HUMAN	7.88E-06	2.271628981	↑ Luminal B HER2 Neg
	Beta-Ala-His dipeptidase	Q96KN2	CNDP1_HUMAN	1.16E-05	1.782534521	↑ Luminal B HER2 Neg
	Lumican	P51884	LUM_HUMAN	1.24E-05	1.753050901	↑ Luminal B HER2 Neg
	Alpha-2-antiplasmin	P08697	A2AP_HUMAN	1.39E-05	1.680683866	↑ Luminal B HER2 Neg
	Biotinidase	P43251	BTD_HUMAN	1.42E-05	1.571319968	↑ Luminal B HER2 Neg
	Immunoglobulin heavy constant alpha 1	P01876	IGHA1_HUMAN	1.81E-05	2.45029046	↑ Luminal B HER2 Neg
	Heparin cofactor 2	P05546	HEP2_HUMAN	2.38E-05	1.568815111	↑ Luminal B HER2 Neg
	Apolipoprotein C-IV	P55056	APOC4_HUMAN	2.43E-05	2.516727604	↑ Luminal B HER2 Neg
	Serum paraoxonase/lactonase 3	Q15166	PON3_HUMAN	3.44E-05	1.614088523	↑ Luminal B HER2 Neg
	Properdin	P27918	PROP_HUMAN	3.87E-05	1.820837643	↑ Luminal B HER2 Neg
	Immunoglobulin kappa constant	P01834	IGKC_HUMAN	6.04E-05	2.234283878	↑ Luminal B HER2 Neg
	Phospholipid transfer protein	P55058	PLTP_HUMAN	0.000127276	1.491697089	↑ Luminal B HER2 Neg
	Cadherin-5	P33151	CADH5_HUMAN	0.000141516	1.802437488	↑ Luminal B HER2 Neg
	Serum paraoxonase/arylesterase 1	P27169	PON1_HUMAN	0.000147602	1.47911577	↑ Luminal B HER2 Neg
	Alpha-2-HS-glycoprotein	P02765	FETUA_HUMAN	0.000221817	1.391954267	↑ Luminal B HER2 Neg
	Immunoglobulin kappa variable 3-11	P04433	KV311_HUMAN	0.000235484	2.727555354	↑ Luminal B HER2 Neg
	Immunoglobulin heavy variable 3-49	A0A0A0MS15	HV349_HUMAN	0.000241713	2.514304893	↑ Luminal B HER2 Neg
	Carboxypeptidase B2	Q96IY4	CBPB2_HUMAN	0.000274145	1.377044298	↑ Luminal B HER2 Neg
	Immunoglobulin kappa variable 6D-21	A0A0A0MT36	KVD21_HUMAN	0.000275144	2.982480226	↑ Luminal B HER2 Neg
	Complement C4-A	P0C0L4	CO4A_HUMAN	0.000311066	1.582264414	↑ Luminal B HER2 Neg
	Immunoglobulin heavy variable 3-9	P01782	HV309_HUMAN	0.000418498	2.709287474	↑ Luminal B HER2 Neg
	Immunoglobulin heavy variable 4-31	P0DP07	HV431_HUMAN	0.000447522	4.063279859	↑ Luminal B HER2 Neg
	Immunoglobulin lambda variable 3-9	A0A075B6K5	LV39_HUMAN	0.000527457	2.179105185	↑ Luminal B HER2 Neg

	Tetranectin	P05452	TETN_HUMAN	0.000529868	1.375681621	↑ Luminal B HER2 Neg
	Alpha-mannosidase 2	Q16706	MA2A1_HUMAN	0.000577191	2.094925838	↑ Luminal B HER2 Neg
	Peroxiredoxin-2	P32119	PRDX2_HUMAN	0.000663743	1.658367127	↑ Luminal B HER2 Neg
	Cholinesterase	P06276	CHLE_HUMAN	0.000739103	2.443938708	↑ Luminal B HER2 Neg
	Immunoglobulin heavy constant gamma 1	P01857	IGHG1_HUMAN	0.000838666	1.832731289	↑ Luminal B HER2 Neg
	Galectin-3-binding protein	Q08380	LG3BP_HUMAN	0.000924525	1.669562645	↑ Luminal B HER2 Neg
	Immunoglobulin lambda variable 1-40	P01703	LV140_HUMAN	0.00099044	2.058275359	↑ Luminal B HER2 Neg
	Kallistatin	P29622	KAIN_HUMAN	0.001372229	1.767108231	↑ Luminal B HER2 Neg
	Alpha-1-acid glycoprotein 1	P02763	A1AG1_HUMAN	0.001636216	2.215227977	↑ Luminal B HER2 Neg
	Apolipoprotein B-100	P04114	APOB_HUMAN	0.001926757	1.618943103	↑ Luminal B HER2 Neg
	Immunoglobulin heavy constant alpha 2	P01877	IGHA2_HUMAN	0.00207989	2.137794634	↑ Luminal B HER2 Neg
	Basement membrane-specific heparan sulfate proteoglycan core protein	P98160	PGBM_HUMAN	0.002185804	2.617271104	↑ Luminal B HER2 Neg
	Cholesteryl ester transfer protein	P11597	CETP_HUMAN	0.002360349	1.663599851	↑ Luminal B HER2 Neg
	Phosphatidylcholine-sterol acyltransferase	P04180	LCAT_HUMAN	0.002466447	2.154281745	↑ Luminal B HER2 Neg
	Immunoglobulin kappa variable 1-16	P04430	KV116_HUMAN	0.002548994	2.530617013	↑ Luminal B HER2 Neg
	Pregnancy zone protein	P20742	PZP_HUMAN	0.002663618	4.343426266	↑ Luminal B HER2 Neg
	Immunoglobulin heavy variable 1-46	P01743	HV146_HUMAN	0.00286485	3.896313023	↑ Luminal B HER2 Neg
	Apolipoprotein A-I	P02647	APOA1_HUMAN	0.002969507	1.292738115	↑ Luminal B HER2 Neg
	Immunoglobulin lambda variable 3-19	P01714	LV319_HUMAN	0.003020851	1.830912469	↑ Luminal B HER2 Neg
	Immunoglobulin heavy variable 1-69D	A0A0B4J2H0	HV69D_HUMAN	0.00307313	2.762019962	↑ Luminal B HER2 Neg
	Apolipoprotein C-III	P02656	APOC3_HUMAN	0.003268869	1.544477322	↑ Luminal B HER2 Neg
	Coagulation factor X	P00742	FA10_HUMAN	0.003928385	1.52489515	↑ Luminal B HER2 Neg
	Pigment epithelium-derived factor	P36955	PEDF_HUMAN	0.004477954	1.582067399	↑ Luminal B HER2 Neg
	Carbonic anhydrase 1	P00915	CAH1_HUMAN	0.004778689	4.78458974	↑ Luminal B HER2 Neg
	Immunoglobulin J chain	P01591	IGJ_HUMAN	0.005504448	1.421634254	↑ Luminal B HER2 Neg
	Immunoglobulin heavy variable 3-74	A0A0B4J1X5	HV374_HUMAN	0.005843078	1.63300774	↑ Luminal B HER2 Neg

	C-reactive protein	P02741	CRP_HUMAN	0.006150605	2.762940453	↑ Luminal B HER2 Neg
	Inter-alpha-trypsin inhibitor heavy chain H4	Q14624	ITIH4_HUMAN	0.006196813	1.442742283	↑ Luminal B HER2 Neg
	Immunoglobulin kappa variable 1-33	P01594	KV133_HUMAN	0.006299022	1.859474241	↑ Luminal B HER2 Neg
	Immunoglobulin lambda-like polypeptide 5	B9A064	IGLL5_HUMAN	0.006474796	1.674164832	↑ Luminal B HER2 Neg
	Immunoglobulin kappa variable 3D-20	A0A0C4DH25	KVD20_HUMAN	0.007387257	1.722873358	↑ Luminal B HER2 Neg
	Lipopolysaccharide-binding protein	P18428	LBP_HUMAN	0.007650788	1.353311199	↑ Luminal B HER2 Neg
	Hemoglobin subunit delta	P02042	HBD_HUMAN	0.007912148	6.029835988	↑ Luminal B HER2 Neg
	Hemoglobin subunit beta	P68871	HBB_HUMAN	0.008912028	36.84859718	↑ Luminal B HER2 Neg
	Hemoglobin subunit alpha	P69905	HBA_HUMAN	0.009565304	24.71644777	↑ Luminal B HER2 Neg
	L-lactate dehydrogenase B chain	P07195	LDHB_HUMAN	0.011088233	1.464826821	↑ Luminal B HER2 Neg
	Thrombospondin-1	P07996	TSP1_HUMAN	0.012439842	2.079938026	↑ Luminal B HER2 Neg
	Serum amyloid P-component	P02743	SAMP_HUMAN	0.013063985	1.786687389	↑ Luminal B HER2 Neg
	Platelet glycoprotein Ib alpha chain	P07359	GP1BA_HUMAN	0.014355443	1.435497779	↑ Luminal B HER2 Neg
	Carbonic anhydrase 2	P00918	CAH2_HUMAN	0.014428974	1.895853685	↑ Luminal B HER2 Neg
	Apolipoprotein C-I	P02654	APOC1_HUMAN	0.016106686	1.456534364	↑ Luminal B HER2 Neg
	Protein AMBP	P02760	AMBP_HUMAN	0.018347785	1.296261299	↑ Luminal B HER2 Neg
	Vitamin K-dependent protein S	P07225	PROS_HUMAN	0.019317019	1.214924135	↑ Luminal B HER2 Neg
	Immunoglobulin heavy variable 1-2	P23083	HV102_HUMAN	0.020170868	1.407214078	↑ Luminal B HER2 Neg
	Mannose-binding protein C	P11226	MBL2_HUMAN	0.021869726	2.267023235	↑ Luminal B HER2 Neg
	Immunoglobulin lambda variable 7-46	A0A075B6I9	LV746_HUMAN	0.023386767	1.309979917	↑ Luminal B HER2 Neg
	Coagulation factor IX	P00740	FA9_HUMAN	0.025068014	1.295436285	↑ Luminal B HER2 Neg
	Apolipoprotein D	P05090	APOD_HUMAN	0.02689663	1.565894604	↑ Luminal B HER2 Neg
	Alpha-1-antichymotrypsin	P01011	AACT_HUMAN	0.027689996	1.834328725	↑ Luminal B HER2 Neg
	Leucine-rich alpha-2-glycoprotein	P02750	A2GL_HUMAN	0.029045042	1.920903764	↑ Luminal B HER2 Neg
	Haptoglobin-related protein	P00739	HPTR_HUMAN	0.029342027	1.312804206	↑ Luminal B HER2 Neg
	Alpha-1-acid glycoprotein 2	P19652	A1AG2_HUMAN	0.029382714	1.963019465	↑ Luminal B HER2 Neg
	Immunoglobulin lambda variable 3-21	P80748	LV321_HUMAN	0.030887039	1.531598796	↑ Luminal B HER2 Neg

	Collectin-11	Q9BWP8	COL11_HUMAN	0.031474412	1.701990672	↑ Luminal B HER2 Neg
	C4b-binding protein beta chain	P20851	C4BPB_HUMAN	0.033906593	1.291393262	↑ Luminal B HER2 Neg
	Mediator of RNA polymerase II transcription subunit 23	Q9ULK4	MED23_HUMAN	0.034189832	1.582094144	↑ Luminal B HER2 Neg
	Immunoglobulin lambda variable 3-25	P01717	LV325_HUMAN	0.037087746	1.366655688	↑ Luminal B HER2 Neg
	Transferrin receptor protein 1	P02786	TFR1_HUMAN	0.0389771	1.371797758	↑ Luminal B HER2 Neg
	Gelsolin	P06396	GELS_HUMAN	0.047520129	1.413658316	↑ Luminal B HER2 Neg
	Immunoglobulin kappa variable 3D-15	A0A087WSY6	KVD15_HUMAN	0.049277542	1.620870123	↑ Luminal B HER2 Neg
	Complement factor D	P00746	CFAD_HUMAN	9.02E-06	5.53018627	↑ Control
	Monocyte differentiation antigen CD14	P08571	CD14_HUMAN	2.51E-05	2.220852523	↑ Control
	Kininogen-1	P01042	KNG1_HUMAN	2.60E-05	2.199628835	↑ Control
	Tenascin-X	P22105	TENX_HUMAN	3.94E-05	1.6826234	↑ Control
	Insulin-like growth factor-binding protein complex acid labile subunit	P35858	ALS_HUMAN	6.12E-05	1.780055952	↑ Control
	Complement factor I	P05156	CFAI_HUMAN	0.000120005	1.759588079	↑ Control
	Serotransferrin	P02787	TRFE_HUMAN	0.000126524	2.02525736	↑ Control
	Serum albumin	P02768	ALBU_HUMAN	0.000171148	1.683767104	↑ Control
	Plasminogen	P00747	PLMN_HUMAN	0.000277629	1.496330117	↑ Control
	Complement factor B	P00751	CFAB_HUMAN	0.000315845	1.866989692	↑ Control
	Complement C3	P01024	CO3_HUMAN	0.000648107	1.956090281	↑ Control
	Hyaluronan-binding protein 2	Q14520	HABP2_HUMAN	0.000924595	1.722448064	↑ Control
	Nuclear receptor coactivator 6	Q14686	NCOA6_HUMAN	0.000998012	1.819162172	↑ Control
	Complement factor H	P08603	CFAH_HUMAN	0.001926344	1.494359684	↑ Control
	Vitamin D-binding protein	P02774	VTDB_HUMAN	0.003402591	1.73672517	↑ Control
	Complement component C8 gamma chain	P07360	CO8G_HUMAN	0.007168406	1.461243032	↑ Control
	Complement factor H-related protein 1	Q03591	FHR1_HUMAN	0.008181812	2.315495477	↑ Control
	Mannan-binding lectin serine protease 2	O00187	MASP2_HUMAN	0.012755497	0.485843644	↑ Control
	Complement factor H-related protein 4	Q92496	FHR4_HUMAN	0.013093998	8.082016269	↑ Control

	Platelet factor 4 variant	P10720	PF4V_HUMAN	0.013149247	8.695958011	↑ Control
	Proteoglycan 4	Q92954	PRG4_HUMAN	0.014358816	1.819998433	↑ Control
	Complement C1q subcomponent subunit A	P02745	C1QA_HUMAN	0.020491258	1.620607626	↑ Control
	Complement C1q subcomponent subunit B	P02746	C1QB_HUMAN	0.020779055	1.40753788	↑ Control
	Apolipoprotein(a)	P08519	APOA_HUMAN	0.027494746	1.753437328	↑ Control
	Extracellular matrix protein 1	Q16610	ECM1_HUMAN	0.028973967	1.594180697	↑ Control
	Complement C1q subcomponent subunit C	P02747	C1QC_HUMAN	0.029229257	1.350965642	↑ Control
	Apolipoprotein M	O95445	APOM_HUMAN	0.033725746	1.267210422	↑ Control
	Coagulation factor XIII B chain	P05160	F13B_HUMAN	0.034419397	1.368179222	↑ Control
	Fibrinogen alpha chain	P02671	FIBA_HUMAN	0.037000628	1.371569583	↑ Control
	Afamin	P43652	AFAM_HUMAN	0.039195834	1.267057624	↑ Control
	Complement C1s subcomponent	P09871	C1S_HUMAN	0.040782815	1.400513823	↑ Control
	Fibronectin	P02751	FINC_HUMAN	0.046347063	1.434387799	↑ Control
**Control *vs.* Luminal B HER2 Positive**	**Protein Name**	**Entry Name**	**UniProt Name**	***p*-value**	**Fold Change**	
	Hemoglobin subunit alpha	P69905	HBA_HUMAN	5.21E-08	3.249069603	↑ Luminal B HER2 Pos
	Hemoglobin subunit beta	P68871	HBB_HUMAN	4.10E-07	3.803126189	↑ Luminal B HER2 Pos
	Pigment epithelium-derived factor	P36955	PEDF_HUMAN	5.54E-07	3.00054601	↑ Luminal B HER2 Pos
	Immunoglobulin heavy variable 3-49	A0A0A0MS15	HV349_HUMAN	1.97E-06	2.863927632	↑ Luminal B HER2 Pos
	Apolipoprotein C-I	P02654	APOC1_HUMAN	2.13E-05	2.739130492	↑ Luminal B HER2 Pos
	Hemoglobin subunit delta	P02042	HBD_HUMAN	2.67E-05	3.306697207	↑ Luminal B HER2 Pos
	Immunoglobulin kappa variable 6D-21	A0A0A0MT36	KVD21_HUMAN	2.68E-05	3.798498309	↑ Luminal B HER2 Pos
	Apolipoprotein C-II	P02655	APOC2_HUMAN	4.39E-05	1.846194109	↑ Luminal B HER2 Pos
	Immunoglobulin lambda variable 1-47	P01700	LV147_HUMAN	5.77E-05	2.850504061	↑ Luminal B HER2 Pos
	Serum amyloid A-4 protein	P35542	SAA4_HUMAN	6.36E-05	2.160815047	↑ Luminal B HER2 Pos
	Immunoglobulin heavy variable 4-31	P0DP07	HV431_HUMAN	7.03E-05	4.89656649	↑ Luminal B HER2 Pos
	Apolipoprotein L1	O14791	APOL1_HUMAN	8.28E-05	1.900750537	↑ Luminal B HER2 Pos
	C4b-binding protein beta chain	P20851	C4BPB_HUMAN	0.00010547	2.155401464	↑ Luminal B HER2 Pos

	Immunoglobulin heavy variable 4-28	A0A0C4DH34	HV428_HUMAN	0.000139308	2.192608342	↑ Luminal B HER2 Pos
	Serum amyloid P-component	P02743	SAMP_HUMAN	0.000181209	2.454265354	↑ Luminal B HER2 Pos
	Immunoglobulin lambda constant 7	A0M8Q6	IGLC7_HUMAN	0.000665214	3.191779846	↑ Luminal B HER2 Pos
	Gelsolin	P06396	GELS_HUMAN	0.000974323	1.951968831	↑ Luminal B HER2 Pos
	Apolipoprotein A-IV	P06727	APOA4_HUMAN	0.001025945	1.544141914	↑ Luminal B HER2 Pos
	Apolipoprotein A-II	P02652	APOA2_HUMAN	0.001171293	1.776849765	↑ Luminal B HER2 Pos
	Plastin-2	P13796	PLSL_HUMAN	0.002018957	4.501473239	↑ Luminal B HER2 Pos
	Kallistatin	P29622	KAIN_HUMAN	0.002130037	1.810271897	↑ Luminal B HER2 Pos
	Polymeric immunoglobulin receptor	P01833	PIGR_HUMAN	0.002912228	1.841450361	↑ Luminal B HER2 Pos
	Apolipoprotein C-III	P02656	APOC3_HUMAN	0.003035205	2.024844997	↑ Luminal B HER2 Pos
	Immunoglobulin lambda variable 3-10	A0A075B6K4	LV310_HUMAN	0.003668828	3.284821215	↑ Luminal B HER2 Pos
	Alpha-1-acid glycoprotein 1	P02763	A1AG1_HUMAN	0.005475644	2.208206222	↑ Luminal B HER2 Pos
	Galectin-3-binding protein	Q08380	LG3BP_HUMAN	0.006686721	1.692847346	↑ Luminal B HER2 Pos
	Complement component C9	P02748	CO9_HUMAN	0.006887579	1.41639914	↑ Luminal B HER2 Pos
	Immunoglobulin lambda variable 3-19	P01714	LV319_HUMAN	0.007858626	1.985710229	↑ Luminal B HER2 Pos
	Immunoglobulin kappa variable 1-13	P0DP09	KV113_HUMAN	0.008612844	2.555407574	↑ Luminal B HER2 Pos
	C-reactive protein	P02741	CRP_HUMAN	0.008678988	3.370377857	↑ Luminal B HER2 Pos
	Complement C4-A	P0C0L4	CO4A_HUMAN	0.010923185	1.488032623	↑ Luminal B HER2 Pos
	Corticosteroid-binding globulin	P08185	CBG_HUMAN	0.011580639	1.994591255	↑ Luminal B HER2 Pos
	Xaa-Pro dipeptidase	P12955	PEPD_HUMAN	0.012031361	2.165189136	↑ Luminal B HER2 Pos
	Immunoglobulin lambda variable 2-11	P01706	LV211_HUMAN	0.01295552	1.570173745	↑ Luminal B HER2 Pos
	Leucine-rich alpha-2-glycoprotein	P02750	A2GL_HUMAN	0.013102391	2.443411846	↑ Luminal B HER2 Pos
	Keratin. type II cytoskeletal 2 epidermal	P35908	K22E_HUMAN	0.01347707	1.499797459	↑ Luminal B HER2 Pos
	Complement component C6	P13671	CO6_HUMAN	0.013979583	2.568266773	↑ Luminal B HER2 Pos
	Cholinesterase	P06276	CHLE_HUMAN	0.015593089	2.140328724	↑ Luminal B HER2 Pos
	Immunoglobulin heavy variable 4-61	A0A0C4DH41	HV461_HUMAN	0.015895648	2.015912108	↑ Luminal B HER2 Pos
	Transthyretin	P02766	TTHY_HUMAN	0.015961518	1.320554649	↑ Luminal B HER2 Pos

	Immunoglobulin lambda variable 1-40	P01703	LV140_HUMAN	0.016785314	1.847853806	↑ Luminal B HER2 Pos
	Immunoglobulin lambda variable 3-9	A0A075B6K5	LV39_HUMAN	0.017837178	1.884838259	↑ Luminal B HER2 Pos
	Complement component C8 alpha chain	P07357	CO8A_HUMAN	0.01851134	1.790235827	↑ Luminal B HER2 Pos
	Immunoglobulin heavy variable 2-70D	A0A0C4DH43	HV70D_HUMAN	0.019383958	2.403651553	↑ Luminal B HER2 Pos
	Plasma protease C1 inhibitor	P05155	IC1_HUMAN	0.0196862	1.276319883	↑ Luminal B HER2 Pos
	Immunoglobulin heavy variable 6-1	A0A0B4J1U7	HV601_HUMAN	0.020191442	1.539338405	↑ Luminal B HER2 Pos
	Alpha-1-antichymotrypsin	P01011	AACT_HUMAN	0.026362641	2.102965709	↑ Luminal B HER2 Pos
	Transferrin receptor protein 1	P02786	TFR1_HUMAN	0.02753498	1.498502068	↑ Luminal B HER2 Pos
	Immunoglobulin lambda variable 3-25	P01717	LV325_HUMAN	0.028817061	1.44474454	↑ Luminal B HER2 Pos
	Serum paraoxonase/arylesterase 1	P27169	PON1_HUMAN	0.029928679	1.297670132	↑ Luminal B HER2 Pos
	Immunoglobulin kappa variable 1-33	P01594	KV133_HUMAN	0.030577751	1.791455428	↑ Luminal B HER2 Pos
	Prenylcysteine oxidase 1	Q9UHG3	PCYOX_HUMAN	0.030672346	1.304466122	↑ Luminal B HER2 Pos
	Vitronectin	P04004	VTNC_HUMAN	0.035879886	1.313506051	↑ Luminal B HER2 Pos
	Immunoglobulin kappa variable 3D-15	A0A087WSY6	KVD15_HUMAN	0.04021589	1.814796476	↑ Luminal B HER2 Pos
	Mannose-binding protein C	P11226	MBL2_HUMAN	0.040341289	2.238378752	↑ Luminal B HER2 Pos
	Immunoglobulin lambda variable 7-46	A0A075B6I9	LV746_HUMAN	0.043520066	1.392118872	↑ Luminal B HER2 Pos
	Zinc-alpha-2-glycoprotein	P25311	ZA2G_HUMAN	0.04421543	1.324950673	↑ Luminal B HER2 Pos
	Apolipoprotein A-I	P02647	APOA1_HUMAN	0.044781205	1.382011988	↑ Luminal B HER2 Pos
	Peroxiredoxin-2	P32119	PRDX2_HUMAN	0.049384389	1.483685429	↑ Luminal B HER2 Pos
	Carboxypeptidase N catalytic chain	P15169	CBPN_HUMAN	0.002104892	1.399185246	↑ Control
	Vitamin K-dependent protein C	P04070	PROC_HUMAN	0.002774626	1.40587544	↑ Control
	Serotransferrin	P02787	TRFE_HUMAN	0.003456816	1.847074609	↑ Control
	Tenascin-X	P22105	TENX_HUMAN	0.008151383	1.459417617	↑ Control
	Histidine-rich glycoprotein	P04196	HRG_HUMAN	0.026419851	1.704325244	↑ Control
	Coagulation factor XIII B chain	P05160	F13B_HUMAN	0.026838663	1.488680992	↑ Control
	Complement C1q subcomponent subunit A	P02745	C1QA_HUMAN	0.038888946	1.704874113	↑ Control
	Complement C1q subcomponent subunit C	P02747	C1QC_HUMAN	0.0443687	1.422681116	↑ Control

**Control *vs.* HER2 Positive**	**Protein Name**	**Entry Name**	**UniProt Name**	***p*-value**	**Fold Change**	
	Complement C5	P01031	CO5_HUMAN	2.95E-15	2.104078338	↑ HER2 Pos
	Clusterin	P10909	CLUS_HUMAN	4.06E-14	2.272392895	↑ HER2 Pos
	Immunoglobulin lambda variable 2-11	P01706	LV211_HUMAN	1.43E-11	2.809437426	↑ HER2 Pos
	Vitronectin	P04004	VTNC_HUMAN	3.15E-11	2.321296178	↑ HER2 Pos
	Hemoglobin subunit delta	P02042	HBD_HUMAN	4.58E-10	5.624944078	↑ HER2 Pos
	Immunoglobulin heavy variable 3-49	A0A0A0MS15	HV349_HUMAN	8.12E-10	4.557168904	↑ HER2 Pos
	Carboxypeptidase N subunit 2	P22792	CPN2_HUMAN	6.65E-09	2.059087817	↑ HER2 Pos
	Adiponectin	Q15848	ADIPO_HUMAN	8.28E-09	9.579128591	↑ HER2 Pos
	Afamin	P43652	AFAM_HUMAN	4.78E-08	2.45261206	↑ HER2 Pos
	Plasminogen	P00747	PLMN_HUMAN	7.26E-08	2.032047844	↑ HER2 Pos
	C4b-binding protein alpha chain	P04003	C4BPA_HUMAN	4.82E-07	2.537213683	↑ HER2 Pos
	Keratin. type I cytoskeletal 9	P35527	K1C9_HUMAN	7.19E-07	2.250911692	↑ HER2 Pos
	Immunoglobulin heavy variable 3-73	A0A0B4J1V6	HV373_HUMAN	1.67E-06	15.87006684	↑ HER2 Pos
	Vitamin D-binding protein	P02774	VTDB_HUMAN	1.96E-06	2.050789715	↑ HER2 Pos
	Coagulation factor XII	P00748	FA12_HUMAN	2.50E-06	4.48323521	↑ HER2 Pos
	Complement C3	P01024	CO3_HUMAN	2.61E-06	2.060113843	↑ HER2 Pos
	Immunoglobulin heavy variable 4-61	A0A0C4DH41	HV461_HUMAN	4.13E-06	5.17636397	↑ HER2 Pos
	Ficolin-2	Q15485	FCN2_HUMAN	7.19E-06	2.289548422	↑ HER2 Pos
	Fibrinogen alpha chain	P02671	FIBA_HUMAN	1.19E-05	1.946313958	↑ HER2 Pos
	Plasma kallikrein	P03952	KLKB1_HUMAN	2.05E-05	2.839283512	↑ HER2 Pos
	Transthyretin	P02766	TTHY_HUMAN	2.53E-05	1.710033019	↑ HER2 Pos
	Hemoglobin subunit alpha	P69905	HBA_HUMAN	3.18E-05	1.915979149	↑ HER2 Pos
	Serum albumin	P02768	ALBU_HUMAN	6.15E-05	1.617918878	↑ HER2 Pos
	Immunoglobulin heavy variable 3-23	P01764	HV323_HUMAN	8.83E-05	2.952483084	↑ HER2 Pos
	Prenylcysteine oxidase 1	Q9UHG3	PCYOX_HUMAN	9.23E-05	2.137601432	↑ HER2 Pos
	Fibronectin	P02751	FINC_HUMAN	0.000101285	1.917381182	↑ HER2 Pos

	Coagulation factor IX	P00740	FA9_HUMAN	0.00012729	1.600811451	↑ HER2 Pos
	Serum amyloid P-component	P02743	SAMP_HUMAN	0.000144697	4.818702495	↑ HER2 Pos
	Complement component C8 alpha chain	P07357	CO8A_HUMAN	0.0001513	1.959771044	↑ HER2 Pos
	Complement factor H	P08603	CFAH_HUMAN	0.000175819	1.83792215	↑ HER2 Pos
	Complement factor H-related protein 4	Q92496	FHR4_HUMAN	0.000204336	3.107198257	↑ HER2 Pos
	Glutathione peroxidase 3	P22352	GPX3_HUMAN	0.000266714	2.917862479	↑ HER2 Pos
	Complement factor D	P00746	CFAD_HUMAN	0.00027027	2.477435187	↑ HER2 Pos
	Immunoglobulin lambda variable 1-51	P01701	LV151_HUMAN	0.000382645	2.419267666	↑ HER2 Pos
	Immunoglobulin heavy variable 3-64	A0A075B6Q5	HV364_HUMAN	0.000401774	2.217759109	↑ HER2 Pos
	Hemoglobin subunit beta	P68871	HBB_HUMAN	0.000414991	2.238927783	↑ HER2 Pos
	Proteoglycan 4	Q92954	PRG4_HUMAN	0.000502566	2.732642249	↑ HER2 Pos
	Selenoprotein P	P49908	SEPP1_HUMAN	0.000539614	3.796988329	↑ HER2 Pos
	Insulin-like growth factor-binding protein complex acid labile subunit	P35858	ALS_HUMAN	0.000815729	1.776314681	↑ HER2 Pos
	Fetuin-B	Q9UGM5	FETUB_HUMAN	0.000972914	2.086049105	↑ HER2 Pos
	Immunoglobulin kappa variable 2D-29	A0A075B6S2	KVD29_HUMAN	0.001323181	2.394042999	↑ HER2 Pos
	Complement component C9	P02748	CO9_HUMAN	0.001535735	1.484378394	↑ HER2 Pos
	Immunoglobulin kappa variable 1D-12	P01611	KVD12_HUMAN	0.00188481	4.471370936	↑ HER2 Pos
	Transferrin receptor protein 1	P02786	TFR1_HUMAN	0.002450216	3.02744249	↑ HER2 Pos
	Cholesteryl ester transfer protein	P11597	CETP_HUMAN	0.00247772	5.150077322	↑ HER2 Pos
	Keratin. type I cytoskeletal 14	P02533	K1C14_HUMAN	0.003456425	2.475906837	↑ HER2 Pos
	Inter-alpha-trypsin inhibitor heavy chain H3	Q06033	ITIH3_HUMAN	0.003797768	1.385787386	↑ HER2 Pos
	Carbonic anhydrase 1	P00915	CAH1_HUMAN	0.0039242	2.696352787	↑ HER2 Pos
	C4b-binding protein beta chain	P20851	C4BPB_HUMAN	0.004369604	1.498125573	↑ HER2 Pos
	Monocyte differentiation antigen CD14	P08571	CD14_HUMAN	0.004529743	1.520207689	↑ HER2 Pos
	Immunoglobulin kappa variable 1D-8	A0A087WSZ0	KVD08_HUMAN	0.005305701	5.877786114	↑ HER2 Pos
	Immunoglobulin lambda variable 3-9	A0A075B6K5	LV39_HUMAN	0.005513194	6.067712275	↑ HER2 Pos
	Thrombospondin-1	P07996	TSP1_HUMAN	0.006951569	2.329232667	↑ HER2 Pos

	Complement component C8 gamma chain	P07360	CO8G_HUMAN	0.00726575	1.510600297	↑ HER2 Pos
	Immunoglobulin lambda variable 5-45	A0A087WSX0	LV545_HUMAN	0.00772078	2.858034161	↑ HER2 Pos
	Immunoglobulin lambda variable 6-57	P01721	LV657_HUMAN	0.009251968	5.252644969	↑ HER2 Pos
	DDB1- and CUL4-associated factor 12-like protein 1	Q5VU92	DC121_HUMAN	0.009802853	58.66867232	↑ HER2 Pos
	Sex hormone-binding globulin	P04278	SHBG_HUMAN	0.009859708	1.968382289	↑ HER2 Pos
	Xaa-Pro dipeptidase	P12955	PEPD_HUMAN	0.010710939	8.299869418	↑ HER2 Pos
	Polymeric immunoglobulin receptor	P01833	PIGR_HUMAN	0.010816257	6.100766211	↑ HER2 Pos
	Keratin type I cytoskeletal 10	P13645	K1C10_HUMAN	0.012361191	1.599012598	↑ HER2 Pos
	C-reactive protein	P02741	CRP_HUMAN	0.013347928	10.58414211	↑ HER2 Pos
	Extracellular matrix protein 1	Q16610	ECM1_HUMAN	0.014298021	1.703726247	↑ HER2 Pos
	Immunoglobulin lambda constant 7	A0M8Q6	IGLC7_HUMAN	0.01446295	9.877133521	↑ HER2 Pos
	Immunoglobulin kappa variable 1-27	A0A075B6S5	KV127_HUMAN	0.014636279	3.663302525	↑ HER2 Pos
	Mannan-binding lectin serine protease 2	O00187	MASP2_HUMAN	0.014654148	2.620003402	↑ HER2 Pos
	Immunoglobulin kappa variable 1-5	P01602	KV105_HUMAN	0.015089737	3.097611266	↑ HER2 Pos
	EGF-containing fibulin-like extracellular matrix protein 1	Q12805	FBLN3_HUMAN	0.015909032	1.962284999	↑ HER2 Pos
	Immunoglobulin heavy variable 2-70D	A0A0C4DH43	HV70D_HUMAN	0.018714547	7.341205699	↑ HER2 Pos
	Immunoglobulin kappa variable 2-24	A0A0C4DH68	KV224_HUMAN	0.019946847	3.792528291	↑ HER2 Pos
	Immunoglobulin heavy constant gamma 2	P01859	IGHG2_HUMAN	0.020013155	1.605775681	↑ HER2 Pos
	Zinc-alpha-2-glycoprotein	P25311	ZA2G_HUMAN	0.020217231	1.455814777	↑ HER2 Pos
	Phosphatidylcholine-sterol acyltransferase	P04180	LCAT_HUMAN	0.020296054	2.819290037	↑ HER2 Pos
	Complement C1r subcomponent	P00736	C1R_HUMAN	0.020917627	1.550066313	↑ HER2 Pos
	Adipocyte plasma membrane-associated protein	Q9HDC9	APMAP_HUMAN	0.021204299	23.89017843	↑ HER2 Pos
	Galectin-3-binding protein	Q08380	LG3BP_HUMAN	0.021587392	1.755243828	↑ HER2 Pos
	Immunoglobulin kappa variable 1D-16	P01601	KVD16_HUMAN	0.023927557	15.36602613	↑ HER2 Pos
	L-selectin	P14151	LYAM1_HUMAN	0.024869054	1.980068571	↑ HER2 Pos
	Immunoglobulin lambda variable 3-10	A0A075B6K4	LV310_HUMAN	0.025145594	7.27762833	↑ HER2 Pos

	Coagulation factor V	P12259	FA5_HUMAN	0.025887503	3.739380413	↑ HER2 Pos
	Matrix metalloproteinase-9	P14780	MMP9_HUMAN	0.026145337	1.695003211	↑ HER2 Pos
	Immunoglobulin heavy variable 1-8	P0DP01	HV108_HUMAN	0.026443384	3.102831151	↑ HER2 Pos
	Complement component C7	P10643	CO7_HUMAN	0.027419082	2.14923558	↑ HER2 Pos
	von Willebrand factor	P04275	VWF_HUMAN	0.02845827	1.636077066	↑ HER2 Pos
	Complement factor H-related protein 1	Q03591	FHR1_HUMAN	0.028584853	1.915607927	↑ HER2 Pos
	Immunoglobulin kappa variable 1-16	P04430	KV116_HUMAN	0.029567938	4.78416667	↑ HER2 Pos
	Cysteine-rich secretory protein 3	P54108	CRIS3_HUMAN	0.034870563	3.338300324	↑ HER2 Pos
	Keratin. type II cytoskeletal 2 epidermal	P35908	K22E_HUMAN	0.035300084	1.805122667	↑ HER2 Pos
	Immunoglobulin heavy variable 3-33	P01772	HV333_HUMAN	0.038394512	8.344444793	↑ HER2 Pos
	Properdin	P27918	PROP_HUMAN	0.038520124	1.768625617	↑ HER2 Pos
	Complement C1q subcomponent subunit A	P02745	C1QA_HUMAN	0.039735285	1.871918365	↑ HER2 Pos
	Pigment epithelium-derived factor	P36955	PEDF_HUMAN	0.042658539	1.861759712	↑ HER2 Pos
	Immunoglobulin heavy variable 1-46	P01743	HV146_HUMAN	0.043543848	4.597946701	↑ HER2 Pos
	Immunoglobulin kappa variable 1-8	A0A0C4DH67	KV108_HUMAN	0.044140149	1.81212033	↑ HER2 Pos
	Immunoglobulin heavy variable 3-72	A0A0B4J1Y9	HV372_HUMAN	0.047935423	1.687200865	↑ HER2 Pos
	Ficolin-3	O75636	FCN3_HUMAN	1.60E-07	5.889667169	↑ Control
	Apolipoprotein A-I	P02647	APOA1_HUMAN	6.44E-07	3.362021938	↑ Control
	Nuclear receptor coactivator 6	Q14686	NCOA6_HUMAN	8.47E-07	8.441443914	↑ Control
	Heparin cofactor 2	P05546	HEP2_HUMAN	1.00E-05	3.661036082	↑ Control
	Kininogen-1	P01042	KNG1_HUMAN	3.73E-05	3.357349607	↑ Control
	Vitamin K-dependent protein S	P07225	PROS_HUMAN	9.45E-05	1.853196173	↑ Control
	Histidine-rich glycoprotein	P04196	HRG_HUMAN	0.000112488	5.940948958	↑ Control
	Immunoglobulin J chain	P01591	IGJ_HUMAN	0.000238757	2.91931338	↑ Control
	N-acetylmuramoyl-L-alanine amidase	Q96PD5	PGRP2_HUMAN	0.000321687	1.799194526	↑ Control
	Angiotensinogen	P01019	ANGT_HUMAN	0.00043286	3.016543479	↑ Control
	Inter-alpha-trypsin inhibitor heavy chain H2	P19823	ITIH2_HUMAN	0.00074408	1.923949583	↑ Control

	Apolipoprotein M	O95445	APOM_HUMAN	0.000850537	1.771464702	↑ Control
	Haptoglobin-related protein	P00739	HPTR_HUMAN	0.000868695	2.12707613	↑ Control
	Alpha-1-antitrypsin	P01009	A1AT_HUMAN	0.001111283	4.959761437	↑ Control
	Inter-alpha-trypsin inhibitor heavy chain H1	P19827	ITIH1_HUMAN	0.001316207	1.682469513	↑ Control
	Beta-2-glycoprotein 1	P02749	APOH_HUMAN	0.001791485	2.272364551	↑ Control
	Alpha-2-HS-glycoprotein	P02765	FETUA_HUMAN	0.001836174	1.586208463	↑ Control
	Apolipoprotein C-IV	P55056	APOC4_HUMAN	0.001950165	6.335750729	↑ Control
	Apolipoprotein A-II	P02652	APOA2_HUMAN	0.002133097	2.248237048	↑ Control
	Trypsin-1	P07477	TRY1_HUMAN	0.002454431	4.217786063	↑ Control
	Apolipoprotein C-III	P02656	APOC3_HUMAN	0.002567875	3.440587523	↑ Control
	Plasma protease C1 inhibitor	P05155	IC1_HUMAN	0.00372715	1.579476004	↑ Control
	Apolipoprotein F	Q13790	APOF_HUMAN	0.005336626	7.55893037	↑ Control
	Carboxypeptidase B2	Q96IY4	CBPB2_HUMAN	0.009929161	1.517845486	↑ Control
	Antithrombin-III	P01008	ANT3_HUMAN	0.018612975	1.308597079	↑ Control
	Complement C1q subcomponent subunit B	P02746	C1QB_HUMAN	0.020285082	1.835321411	↑ Control
	Inter-alpha-trypsin inhibitor heavy chain H4	Q14624	ITIH4_HUMAN	0.02171345	1.978217456	↑ Control
	Alpha-2-macroglobulin	P01023	A2MG_HUMAN	0.021759431	1.818611048	↑ Control
	Ceruloplasmin	P00450	CERU_HUMAN	0.023271461	3.101927554	↑ Control
	Alpha-1B-glycoprotein	P04217	A1BG_HUMAN	0.030481015	3.465086804	↑ Control
	Haptoglobin	P00738	HPT_HUMAN	0.032119775	1.834688417	↑ Control
	Serum amyloid A-4 protein	P35542	SAA4_HUMAN	0.033032185	2.177960496	↑ Control
	Complement C1q subcomponent subunit C	P02747	C1QC_HUMAN	0.034179123	1.548970133	↑ Control
	Serotransferrin	P02787	TRFE_HUMAN	0.034307125	1.559639179	↑ Control
	Apolipoprotein(a)	P08519	APOA_HUMAN	0.046687987	2.207374657	↑ Control
***vs.* Triple Negativ**	**Protein Name**	**Entry Name**	**UniProt Name**	***p*-value**	**Fold Change**	
	Immunoglobulin heavy variable 4-31	P0DP07	HV431_HUMAN	3.40E-06	6.336923351	↑ Triple Negative
	Hemoglobin subunit beta	P68871	HBB_HUMAN	4.66E-06	1.856970774	↑ Triple Negative

	Beta-2-glycoprotein 1	P02749	APOH_HUMAN	9.79E-06	1.826994929	↑ Triple Negative
	Haptoglobin	P00738	HPT_HUMAN	1.31E-05	2.047411407	↑ Triple Negative
	Coagulation factor IX	P00740	FA9_HUMAN	1.45E-05	1.878304045	↑ Triple Negative
	Apolipoprotein C-I	P02654	APOC1_HUMAN	1.84E-05	2.981683921	↑ Triple Negative
	Complement component C9	P02748	CO9_HUMAN	2.46E-05	2.09824133	↑ Triple Negative
	Afamin	P43652	AFAM_HUMAN	2.61E-05	1.741122003	↑ Triple Negative
	Galectin-3-binding protein	Q08380	LG3BP_HUMAN	2.64E-05	2.278337206	↑ Triple Negative
	Hemoglobin subunit delta	P02042	HBD_HUMAN	2.86E-05	6.391315854	↑ Triple Negative
	C4b-binding protein beta chain	P20851	C4BPB_HUMAN	2.98E-05	1.75486955	↑ Triple Negative
	Lipopolysaccharide-binding protein	P18428	LBP_HUMAN	4.09E-05	3.179730825	↑ Triple Negative
	Immunoglobulin heavy variable 3-49	A0A0A0MS15	HV349_HUMAN	4.10E-05	5.091742669	↑ Triple Negative
	Apolipoprotein C-IV	P55056	APOC4_HUMAN	4.12E-05	2.359368787	↑ Triple Negative
	Polymeric immunoglobulin receptor	P01833	PIGR_HUMAN	4.79E-05	2.533797187	↑ Triple Negative
	Immunoglobulin lambda variable 1-47	P01700	LV147_HUMAN	5.70E-05	2.588797941	↑ Triple Negative
	Dopamine beta-hydroxylase	P09172	DOPO_HUMAN	0.000148225	3.155747162	↑ Triple Negative
	Plasma protease C1 inhibitor	P05155	IC1_HUMAN	0.000185866	1.444277794	↑ Triple Negative
	Serum amyloid P-component	P02743	SAMP_HUMAN	0.000323991	2.653614489	↑ Triple Negative
	Zinc-alpha-2-glycoprotein	P25311	ZA2G_HUMAN	0.000734893	1.658606922	↑ Triple Negative
	Serum amyloid A-4 protein	P35542	SAA4_HUMAN	0.001564521	1.767558789	↑ Triple Negative
	Vitronectin	P04004	VTNC_HUMAN	0.001792262	1.453911285	↑ Triple Negative
	Keratin. type I cytoskeletal 9	P35527	K1C9_HUMAN	0.001912466	1.575612234	↑ Triple Negative
	C-reactive protein	P02741	CRP_HUMAN	0.002038334	7.916907504	↑ Triple Negative
	Retinol-binding protein 4	P02753	RET4_HUMAN	0.002148466	1.436880465	↑ Triple Negative
	Pigment epithelium-derived factor	P36955	PEDF_HUMAN	0.003263088	2.30578555	↑ Triple Negative
	Hemoglobin subunit alpha	P69905	HBA_HUMAN	0.003339445	1.715827887	↑ Triple Negative
	von Willebrand factor	P04275	VWF_HUMAN	0.003416651	1.583518619	↑ Triple Negative
	Protein AMBP	P02760	AMBP_HUMAN	0.004092418	1.786125711	↑ Triple Negative

	Complement factor I	P05156	CFAI_HUMAN	0.004267426	1.531161402	↑ Triple Negative
	Apolipoprotein E	P02649	APOE_HUMAN	0.005108321	1.297484301	↑ Triple Negative
	Peroxiredoxin-2	P32119	PRDX2_HUMAN	0.005462251	1.56831661	↑ Triple Negative
	IgGFc-binding protein	Q9Y6R7	FCGBP_HUMAN	0.006429638	1.543604283	↑ Triple Negative
	Transferrin receptor protein 1	P02786	TFR1_HUMAN	0.006576023	2.659048849	↑ Triple Negative
	Complement component C6	P13671	CO6_HUMAN	0.006913895	3.211027998	↑ Triple Negative
	Matrix metalloproteinase-9	P14780	MMP9_HUMAN	0.007583113	2.1547884	↑ Triple Negative
	Immunoglobulin heavy variable 1-8	P0DP01	HV108_HUMAN	0.007621133	2.188587398	↑ Triple Negative
	Gelsolin	P06396	GELS_HUMAN	0.008449427	1.600858701	↑ Triple Negative
	Cadherin-5	P33151	CADH5_HUMAN	0.008460741	1.74346858	↑ Triple Negative
	Plasma serine protease inhibitor	P05154	IPSP_HUMAN	0.008634823	1.750498274	↑ Triple Negative
	Immunoglobulin heavy variable 1-2	P23083	HV102_HUMAN	0.008919673	1.559197168	↑ Triple Negative
	Fetuin-B	Q9UGM5	FETUB_HUMAN	0.009781609	1.67811206	↑ Triple Negative
	Voltage-dependent L-type calcium channel subunit alpha-1F	O60840	CAC1F_HUMAN	0.010760078	3.330169664	↑ Triple Negative
	Properdin	P27918	PROP_HUMAN	0.010990918	2.859498176	↑ Triple Negative
	Heparin cofactor 2	P05546	HEP2_HUMAN	0.011416361	1.356715555	↑ Triple Negative
	Immunoglobulin lambda variable 1-40	P01703	LV140_HUMAN	0.011566545	2.233258232	↑ Triple Negative
	Transthyretin	P02766	TTHY_HUMAN	0.012616261	1.557894268	↑ Triple Negative
	Apolipoprotein A-IV	P06727	APOA4_HUMAN	0.013273745	1.393597405	↑ Triple Negative
	Immunoglobulin kappa variable 2D-29	A0A075B6S2	KVD29_HUMAN	0.014548683	1.976553582	↑ Triple Negative
	Immunoglobulin lambda constant 7	A0M8Q6	IGLC7_HUMAN	0.015204337	2.30606418	↑ Triple Negative
	Hyaluronan-binding protein 2	Q14520	HABP2_HUMAN	0.017778041	1.837364492	↑ Triple Negative
	Complement component C7	P10643	CO7_HUMAN	0.018680554	2.247802867	↑ Triple Negative
	Immunoglobulin lambda variable 3-9	A0A075B6K5	LV39_HUMAN	0.018777301	5.992460294	↑ Triple Negative
	Apolipoprotein C-III	P02656	APOC3_HUMAN	0.020727463	1.580436985	↑ Triple Negative
	Complement factor H-related protein 4	Q92496	FHR4_HUMAN	0.021147868	2.333186411	↑ Triple Negative

	Hemopexin	P02790	HEMO_HUMAN	0.021507045	1.152784299	↑ Triple Negative
	Apolipoprotein C-II	P02655	APOC2_HUMAN	0.022665952	1.368769534	↑ Triple Negative
	Cathelicidin antimicrobial peptide	P49913	CAMP_HUMAN	0.024037233	2.485659851	↑ Triple Negative
	Keratin. type II cytoskeletal 2 epidermal	P35908	K22E_HUMAN	0.024080471	1.556563063	↑ Triple Negative
	Complement C2	P06681	CO2_HUMAN	0.024115534	1.265184448	↑ Triple Negative
	Keratin. type II cytoskeletal 1	P04264	K2C1_HUMAN	0.02492244	1.394646766	↑ Triple Negative
	Xaa-Pro dipeptidase	P12955	PEPD_HUMAN	0.025128744	8.197509777	↑ Triple Negative
	Sex hormone-binding globulin	P04278	SHBG_HUMAN	0.025579843	2.464769888	↑ Triple Negative
	Tetranectin	P05452	TETN_HUMAN	0.026202789	1.352737436	↑ Triple Negative
	Immunoglobulin heavy variable 4-30-2	A0A087WSY4	HV432_HUMAN	0.028590349	4.459516925	↑ Triple Negative
	Keratin type I cytoskeletal 14	P02533	K1C14_HUMAN	0.030094113	1.561799843	↑ Triple Negative
	Immunoglobulin lambda variable 3-10	A0A075B6K4	LV310_HUMAN	0.031988087	3.907325632	↑ Triple Negative
	Lumican	P51884	LUM_HUMAN	0.032174228	1.406284475	↑ Triple Negative
	Clusterin	P10909	CLUS_HUMAN	0.03243292	1.190567099	↑ Triple Negative
	Histidine-rich glycoprotein	P04196	HRG_HUMAN	0.032443596	1.394921042	↑ Triple Negative
	Attractin	O75882	ATRN_HUMAN	0.033317422	1.248304377	↑ Triple Negative
	Immunoglobulin kappa variable 2D-30	A0A075B6S6	KVD30_HUMAN	0.035772725	1.512873113	↑ Triple Negative
	Alpha-1-acid glycoprotein 2	P19652	A1AG2_HUMAN	0.035780803	2.040647761	↑ Triple Negative
	Immunoglobulin kappa variable 1-13	P0DP09	KV113_HUMAN	0.035987663	3.049898703	↑ Triple Negative
	Kallistatin	P29622	KAIN_HUMAN	0.038988076	1.506271081	↑ Triple Negative
	Immunoglobulin kappa variable 1-6	A0A0C4DH72	KV106_HUMAN	0.039260496	1.537751007	↑ Triple Negative
	Immunoglobulin kappa variable 1D-8	A0A087WSZ0	KVD08_HUMAN	0.040751298	8.357751705	↑ Triple Negative
	Immunoglobulin heavy variable 7-4-1	A0A0J9YVY3	HV741_HUMAN	0.040865385	2.222501572	↑ Triple Negative
	Prenylcysteine oxidase 1	Q9UHG3	PCYOX_HUMAN	0.041567545	1.458990938	↑ Triple Negative
	Glutathione peroxidase 3	P22352	GPX3_HUMAN	0.043164541	4.984894872	↑ Triple Negative
	C4b-binding protein alpha chain	P04003	C4BPA_HUMAN	0.044164801	1.446279607	↑ Triple Negative
	DDB1- and CUL4-associated factor 12-like	Q5VU92	DC121_HUMAN	0.047731266	29.59481864	↑ Triple Negative

	protein 1					
	Complement C4-A	P0C0L4	CO4A_HUMAN	0.048405342	1.576071914	↑ Triple Negative
	Monocyte differentiation antigen CD14	P08571	CD14_HUMAN	0.04850195	1.325673944	↑ Triple Negative
	Carbonic anhydrase 2	P00918	CAH2_HUMAN	0.048684224	2.001285154	↑ Triple Negative
	Cholesteryl ester transfer protein	P11597	CETP_HUMAN	0.049353744	2.914758788	↑ Triple Negative
	Platelet basic protein	P02775	CXCL7_HUMAN	0.04971791	23.75806076	↑ Triple Negative
	Serotransferrin	P02787	TRFE_HUMAN	0.010845697	1.589906375	↑ Control
	Complement C1q subcomponent subunit C	P02747	C1QC_HUMAN	0.011358029	1.543819449	↑ Control
	CD5 antigen-like	O43866	CD5L_HUMAN	0.012543111	1.999836008	↑ Control
	L-selectin	P14151	LYAM1_HUMAN	0.013557058	1.42160042	↑ Control

Table S4. Specific or unique differentially expressed proteins (up-regulated and down-regulated) (p-value ≤ 0.05) found in the protein patterns of the *ex vivo* formed coronas after the analysis by SWATH-MS for the different breast cancer subtypes (LA, n = 11; LB-, n = 10; LB+, n = 7; HER2+, n = 6; TNBC, n = 8) in comparison with healthy control (HC) samples.

**Luminal A** *Entry name* Statistically significant		**Luminal B-HER negative** *Entry name* Statistically significant			**Luminal B-HER positive** *Entry name* Statistically significant			**HER2+** *Entry name* Statistically significant			**TNBC** *Entry name* Statistically significant		
up-regulated n = 42	down-regulated n = 18	up-regulated n = 100		down-regulated n = 32	up-regulated n = 59		down-regulated n = 8	up-regulated n = 95		down-regulated n = 35	up-regulated n = 87		down-regulated n = 4
uniques = 4	uniques = 4	uniques = 25		uniques = 2	uniques = 2		uniques = 0	uniques = 23		uniques = 5	uniques = 9		uniques = 1
A2AP ↑	A2MG ↓	A1AG1 ↑	IGHA1 ↑	AFAM ↓	A1AG1 ↑	K22E ↑	C1QA ↓	ADIPO ↑	HV461 ↑	A1AT ↓	A1AG2 ↑	HV102 ↑	C1QC ↓
**APOC3 ↑**	CADH5 ↓	A1AG2 ↑	IGHA2 ↑	ALBU ↓	A2GL ↑	KAIN ↑	C1QC ↓	AFAM ↑	HV70D ↑	A1BG ↓	AFAM ↑	HV108 ↑	CD5L ↓
APOH ↑	CBG ↓	A1BG ↑	IGHG1 ↑	ALS ↓	AACT ↑	KV113 ↑	CBPN ↓	ALBU ↑	IGHG2 ↑	A2MG ↓	AMBP ↑	**HV349 ↑**	LYAM1 ↓
APOL1 ↑	CBPN ↓	A2AP ↑	IGJ ↑	APOA ↓	APOA1 ↑	KV133 ↑	F13B ↓	ALS ↑	IGLC7 ↑	ANGT ↓	APOA4 ↑	HV431 ↑	**TRFE ↓**
C1R ↑	CERU ↓	A2GL ↑	IGKC↑	APOM ↓	APOA2 ↑	KVD15 ↑	HRG ↓	APMAP ↑	ITIH3 ↑	ANT3 ↓	APOC1↑	HV432 ↑	
C1RL ↑	CNDP1 ↓	AACT ↑	IGLC7 ↑	C1QA ↓	APOA4 ↑	KVD21 ↑	PROC ↓	C1QA ↑	K1C10 ↑	APOA ↓	APOC2 ↑	HV741 ↑	
C4BPA ↑	CPN2 ↓	AMBP ↑	IGLL5 ↑	C1QB ↓	APOC1 ↑	LG3BP ↑	TENX ↓	C1R ↑	K1C14 ↑	APOA1 ↓	APOC3 ↑	IC1 ↑	
CAMP ↑	HV124 ↓	ANGT ↑	ITIH1 ↑	C1QC↓	APOC2 ↑	LV140 ↑	**TRFE ↓**	C4BPA ↑	K1C9 ↑	APOA2 ↓	APOC4 ↑	IGLC7 ↑	
CFAB ↑	**HV349 ↓**	APOA1 ↑	ITIH2 ↑	C1S ↓	**APOC3 ↑**	LV147 ↑		C4BPB ↑	K22E ↑	**APOC3 ↓**	APOE ↑	IPSP ↑	
CO8B ↑	HV372 ↓	APOA2 ↑	ITIH4 ↑	CD14 ↓	APOL1 ↑	LV211 ↑		CAH1 ↑	KLKB1 ↑	APOC4 ↓	APOH ↑	K1C14 ↑	
CO8G ↑	HV428 ↓	APOB ↑	KAIN ↑	CFAB ↓	C4BPB ↑	LV310 ↑		CD14 ↑	KV105 ↑	APOF ↓	ATRN ↑	K1C9 ↑	
CO9 ↑	HV741 ↓	APOC1 ↑	KV116 ↑	CFAD ↓	CBG ↑	LV319 ↑		CETP ↑	KV108 ↑	APOH ↓	C4BPA ↑	K22E ↑	
COL11 ↑	IGHM ↓	APOC2 ↑	KV133 ↑	CFAH ↓	CHLE ↑	LV325 ↑		CFAD ↑	KV116 ↑	APOM ↓	C4BPB ↑	K2C1 ↑	
**CRP ↑**	K1C14 ↓	**APOC3 ↑**	KV311 ↑	CFAI ↓	CO4A ↑	LV39 ↑		CFAH ↑	KV127 ↑	C1QB ↓	CAC1F ↑	KAIN ↑	
DOPO ↑	KV108 ↓	APOC4 ↑	KV401 ↑	CO3 ↓	CO6 ↑	LV746		CLUS ↑	KV224 ↑	C1QC ↓	CADH5 ↑	KV106 ↑	
FA10 ↑	KV320 ↓	APOD ↑	KVD15 ↑	CO8G ↓	CO8A ↑	MBL2 ↑		CO3 ↑	KVD08 ↑	CBPB2 ↓	CAH2 ↑	KV113 ↑	
FA9 ↑	SHBG ↓	APOL1 ↑	KVD20 ↑	ECM1↓	CO9 ↑	PCYOX ↑		CO5 ↑	KVD12 ↑	CERU ↓	CAMP ↑	KVD08 ↑	
FCGBP ↑	ZPI ↓	BTD ↑	KVD21↑	F13B ↓	**CRP ↑**	PEDF ↑		CO7 ↑	KVD16 ↑	FCN3 ↓	CD14 ↑	KVD29 ↑	
FCN2 ↑		C4BPB ↑	LBP ↑	FHR1 ↓	GELS	PEPD ↑		CO8A ↑	KVD29 ↑	FETUA ↓	CETP ↑	KVD30 ↑	
FCN3 ↑		CADH5 ↑	LCAT ↑	FHR4 ↓	HBA ↑	PIGR ↑		CO8G ↑	LCAT ↑	HEP2 ↓	CFAI ↑	LBP ↑	
FHR2 ↑		CAH1 ↑	LDHB ↑	FIBA ↓	**HBB ↑**	PLSL ↑		CO9 ↑	LG3BP ↑	HPT ↓	CLUS ↑	LG3BP ↑	
FIBA ↑		CAH2 ↑	LG3BP ↑	FINC ↓	HBD ↑	PON1 ↑		CPN2 ↑	LV151 ↑	HPTR ↓	CO2 ↑	LUM ↑	
FINC ↑		CAMP ↑	LUM ↑	HABP2 ↓	**HV349 ↑**	PRDX2 ↑		CRIS3 ↑	LV211 ↑	HRG ↓	CO4A ↑	LV140 ↑	

HABP2 ↑		CBPB2 ↑	LV140 ↑	KNG1↓	HV428 ↑	**SAA4 ↑**		**CRP ↑**	LV310 ↑	IC1 ↓	CO6 ↑	LV147 ↑	
**HBB ↑**		CETP ↑	LV147 ↑	MASP2 ↓	HV431 ↑	**SAMP ↑**		DC121 ↑	LV39 ↑	IGJ ↓	CO7 ↑	LV310 ↑	
HEMO ↑		CHLE ↑	LV223 ↑	NCOA6 ↓	HV461 ↑	TFR1 ↑		ECM1 ↑	LV545 ↑	ITIH1 ↓	CO9 ↑	LV39 ↑	
HPT ↑		CNDP1↑	LV310 ↑	PF4V ↓	HV601↑	TTHY ↑		FA12 ↑	LV657 ↑	ITIH2 ↓	**CRP ↑**	MMP9 ↑	
HRG ↑		CO4A ↑	LV319 ↑	PLMN ↓	HV70D ↑	VTNC ↑		FA5 ↑	LYAM1 ↑	ITIH4 ↓	CXCL7 ↑	PCYOX ↑	
IC1 ↑		CO6 ↑	LV321 ↑	PRG4 ↓	IC1 ↑	ZA2G ↑		FA9 ↑	MASP2 ↑	KNG1 ↓	DC121 ↑	PEDF ↑	
IPSP ↑		COL11 ↑	LV325 ↑	TENX ↓	IGLC7 ↑			FBLN3 ↑	MMP9 ↑	NCOA6 ↓	DOPO ↑	PEPD ↑	
ITIH3 ↑		**CRP ↑**	LV39 ↑	**TRFE ↓**				FCN2 ↑	PCYOX ↑	PGRP2 ↓	FA9 ↑	PIGR ↑	
LAMP2 ↑		FA10 ↑	LV746 ↑	VTDB ↓				FETUB ↑	PEDF ↑	PROS ↓	FCGBP ↑	PRDX2 ↑	
LBP ↑		FA9 ↑	MA2A1 ↑					FHR1 ↑	PEPD ↑	**SAA4 ↓**	FETUB ↑	PROP ↑	
NCOA6 ↑		FCGBP ↑	MBL2 ↑					FHR4 ↑	PIGR ↑	**TRFE ↓**	FHR4 ↑	RET4 ↑	
PLMN ↑		FETUA ↑	MED23 ↑					FIBA ↑	PLMN ↑	TRY1 ↓	GELS ↑	**SAA4 ↑**	
PRDX2 ↑		GELS ↑	PEDF ↑					FINC ↑	PRG4 ↑		GPX3 ↑	**SAMP ↑**	
PROC ↑		GP1BA ↑	PGBM ↑					GPX3 ↑	PROP ↑		HABP2 ↑	SHBG ↑	
PROS ↑		HBA ↑	PLTP ↑					HBA ↑	**SAMP ↑**		HBA ↑	TETN ↑	
**SAA4 ↑**		**HBB ↑**	PON1 ↑					**HBB ↑**	SEPP1 ↑		**HBB ↑**	TFR1 ↑	
**SAMP ↑**		HBD ↑	PON3 ↑					HBD ↑	SHBG ↑		HBD ↑	TTHY ↑	
**TRFE ↑**		HEP2 ↑	PRDX2 ↑					HV108 ↑	TFR1 ↑		HEMO ↑	VTNC ↑	
VTNC ↑		HPTR ↑	PROP ↑					HV146 ↑	TSP1 ↑		HEP2 ↑	VWF ↑	
		HV102 ↑	PROS ↑					HV323 ↑	TTHY ↑		HPT ↑	ZA2G ↑	
		HV146 ↑	PZP ↑					HV333 ↑	VTDB ↑		HRG ↑		
		HV309 ↑	RET4 ↑					**HV349 ↑**	VTNC ↑				
		**HV349 ↑**	**SAA4 ↑**					HV364 ↑	VWF ↑				
		HV353 ↑	**SAMP ↑**					HV372 ↑	ZA2G ↑				
		HV374 ↑	TETN ↑					HV373 ↑					
		HV431 ↑	TFR1 ↑										
		HV69D ↑	TSP1 ↑										

Table S5. Differentially expressed proteins (up-regulated and down-regulated) (p-value ≤ 0.05) found in the protein patterns of the *ex vivo* formed coronas after the analysis by SWATH-MS common and specific for the different breast cancer subtypes (LA, n = 11; LB-, n = 10; LB+, n = 7; HER2+, n = 6; TNBC, n = 8) in comparison with controls samples. The accession number, gene name and species (Human) were reported.

Protein Name	UniProt Name	Entry Name	Gene	Luminal A	Luminal B HER2 Negative	Luminal B HER2 Positive	HER2 Positive	Triple Negative
**Serum amyloid P-component Fold Change**	**SAMP_HUMAN**	**P02743**	**APCS**	**X** 2.37	**X** 1.79	**X** 2.45	**X** 4.82	**X** 2.65
**C-reactive protein Fold Change**	**CRP_HUMAN**	**P02741**	**CRP**	**X** 3.79	**X** 2.76	**X** 3.37	**X** 10.58	**X** 7.92
**Hemoglobin subunit beta Fold Change**	**HBB_HUMAN**	**P68871**	**HBB**	**X** 1.42	**X** 36.85	**X** 3.80	**X** 2.39	**X** 1.85
**Serotransferrin**	**TRFE_HUMAN**	**P02787**	**TF**	**X** 1.30	**X** 2.02	**X** 1.85	**X** 1.56	**X** 1.59
**Immunoglobulin heavy variable 3-49**	**HV349_HUMAN**	**A0A0A0MS 15**	**IGHV3- 49**	**X** 1.74	**X** 2.51	**X** 2.86	**X** 4.56	**X** 5.09
**Apolipoprotein C-III**	**APOC3_HUMA N**	**P02656**	**APOC3**	**X** 1.70	**X** 1.54	**X** 2.02	**X**	**X** 1.85
**Serum amyloid A-4 protein**	**SAA4_HUMAN**	**P35542**	**SAA4**	**X** 1.46	**X** 2.24	**X** 2.16	**X** 2.39	**X** 1.85
Complement C1r subcomponent-like protein	C1RL_HUMAN	Q9NZP8	C1RL	X				

Complement component C8 beta chain	CO8B_HUMAN	P07358	C8B	X				
Complement factor H-related protein 2	FHR2_HUMAN	P36980	CFHR2	X				
Lysosome-associated membrane glycoprotein 2	LAMP2_HUMAN	P13473	LAMP2	X				
Immunoglobulin heavy variable 1-24	HV124_HUMAN	A0A0C4DH33	IGHV1-24	X				
Immunoglobulin heavy constant mu	IGHM_HUMAN	P01871	IGHM	X				
Immunoglobulin kappa variable 3-20	KV320_HUMAN	P01619	IGKV3-20	X				
Protein Z-dependent protease inhibitor	ZPI_HUMAN	Q9UK55	SERPINA10	X				
Apolipoprotein B-100	APOB_HUMAN	P04114	APOB		X			
Apolipoprotein D	APOD_HUMAN	P05090	APOD		X			
Biotinidase	BTD_HUMAN	P43251	BTD		X			
Platelet glycoprotein Ib alpha chain	GP1BA_HUMAN	P07359	GP1BA		X			
Immunoglobulin heavy variable 3-9	HV309_HUMAN	P01782	IGHV3-9		X			
Immunoglobulin heavy variable 3-53	HV353_HUMAN	P01767	IGHV3-53		X			
Immunoglobulin heavy variable 3-74	HV374_HUMAN	A0A0B4J1X5	IGHV3-74		X			
Immunoglobulin heavy variable 1-69D	HV69D_HUMAN	A0A0B4J2H0	IGHV1-69D		X			
Immunoglobulin heavy constant alpha 1	IGHA1_HUMAN	P01876	IGHA1		X			
Basement membrane-specific	IGHA2_HUMAN	P01877	IGHA2		X			

heparan sulfate proteoglycan core protein								
Immunoglobulin heavy constant gamma 1	IGHG1_HUMAN	P01857	IGHG1		X			
Immunoglobulin kappa constant	IGKC_HUMAN	P01834	IGKC		X			
Immunoglobulin lambda-like polypeptide 5	IGLL5_HUMAN	B9A064	IGLL5		X			
Immunoglobulin kappa variable 3-11	KV311_HUMAN	P04433	IGKV3-11		X			
Immunoglobulin kappa variable 4-1	KV401_HUMAN	P06312	IGKV4-1		X			
Immunoglobulin kappa variable 3D-20	KVD20_HUMAN	A0A0C4DH25	IGKV3D-20		X			
L-lactate dehydrogenase B chain	LDHB_HUMAN	P07195	LDHB		X			
Immunoglobulin lambda variable 2-23	LV223_HUMAN	P01705	IGLV2-23		X			
Immunoglobulin lambda variable 3-21	LV321_HUMAN	P80748	IGLV3-21		X			
Alpha-mannosidase 2	MA2A1_HUMAN	Q16706	MAN2A1		X			
Mediator of RNA polymerase II transcription subunit 23	MED23_HUMAN	Q9ULK4	MED23		X			
Basement membrane-specific heparan sulfate proteoglycan core protein	PGBM_HUMAN	P98160	HSPG2		X			
Phospholipid transfer protein	PLTP_HUMAN	P55058	PLTP		X			
Serum paraoxonase/lactonase 3	PON3_HUMAN	Q15166	PON3		X			
Pregnancy zone protein	PZP_HUMAN	P20742	PZP		X			
Complement C1s subcomponent	C1S_HUMAN	P09871	C1S		X			

Platelet factor 4 variant	PF4V_HUMAN	P10720	PF4V1		X			
Immunoglobulin heavy variable 6-1	HV601_HUMAN	A0A0B4J1U7	IGHV6-1			X		
Plastin-2	PLSL_HUMAN	P13796	LCP1			X		
Adiponectin	ADIPO_HUMAN	Q15848	ADIPOQ				X	
Immunoglobulin heavy variable 3-64	APMAP_HUMAN	Q9HDC9	APMAP				X	
Complement C5	CO5_HUMAN	P01031	C5				X	
Cysteine-rich secretory protein 3	CRIS3_HUMAN	P54108	CRISP3				X	
Coagulation factor XII	FA12_HUMAN	P00748	F12				X	
Coagulation factor V	FA5_HUMAN	P12259	F5				X	
EGF-containing fibulin-like extracellular matrix protein 1	FBLN3_HUMAN	Q12805	EFEMP1				X	
Immunoglobulin heavy variable 3-23	HV323_HUMAN	P01764	IGHV3-23				X	
Immunoglobulin heavy variable 3-33	HV333_HUMAN	P01772	IGHV3-33				X	
Immunoglobulin heavy variable 3-64	HV364_HUMAN	A0A075B6Q5	IGHV3-64				X	
Immunoglobulin heavy variable 3-73	HV373_HUMAN	A0A0B4J1V6	IGHV3-73				X	
Immunoglobulin heavy constant gamma 2	IGHG2_HUMAN	P01859	IGHG2				X	
Keratin, type I cytoskeletal 10	K1C10_HUMAN	P13645	KRT10				X	
Plasma kallikrein	KLKB1_HUMAN	P03952	KLKB1				X	
Immunoglobulin kappa variable 1-5	KV105_HUMAN	P01602	IGKV1-5				X	
Immunoglobulin kappa variable 1-27	KV127_HUMAN	A0A075B6S5	IGKV1-27				X	
Immunoglobulin kappa variable 2-24	KV224_HUMAN	A0A0C4DH68	IGKV2-24				X	

Immunoglobulin kappa variable 1D-12	KVD12_HUMAN	P01611	IGKV1D-12				X	
Immunoglobulin kappa variable 1D-16	KVD16_HUMAN	P01601	IGKV1D-16				X	
Immunoglobulin lambda variable 1-51	LV151_HUMAN	P01701	IGLV1-51				X	
Immunoglobulin lambda variable 5-45	LV545_HUMAN	A0A087WSX0	IGLV5-45				X	
Immunoglobulin lambda variable 6-57	LV657_HUMAN	P01721	IGLV6-57				X	
Selenoprotein P	SEPP1_HUMAN	P49908	SELENOP				X	
Alpha-1-antitrypsin	A1AT_HUMAN	P01009	SERPINA1				X	
Antithrombin-III	ANT3_HUMAN	P01008	SERPINC1				X	
Apolipoprotein F	APOF_HUMAN	Q13790	APOF				X	
N-acetylmuramoyl-L-alanine amidase	PGRP2_HUMAN	Q96PD5	PGLYRP2				X	
Trypsin-1	TRY1_HUMAN	P07477	PRSS1				X	
Apolipoprotein E	APOE_HUMAN	P02649	APOE					X
Attractin	ATRN_HUMAN	O75882	ATRN					X
Voltage-dependent L-type calcium channel subunit alpha-1F	CAC1F_HUMAN	O60840	CACNA1F					X
Complement C2	CO2_HUMAN	P06681	C2					X
Platelet basic protein	CXCL7_HUMAN	P02775	PPBP					X
Immunoglobulin heavy variable 4-30-2	HV432_HUMAN	A0A087WSY4	IGHV4-30-2					X
Keratin, type II cytoskeletal 1	K2C1_HUMAN	P04264	KRT1					X
Immunoglobulin kappa variable 1-6	KV106_HUMAN	A0A0C4DH72	IGKV1-6					X

Immunoglobulin kappa variable 2D-30	KVD30_HUMAN	A0A075B6S6	IGKV2D-30					X
CD5 antigen-like	CD5L_HUMAN	O43866	CD5L					X
Peroxiredoxin-2	PRDX2_HUMAN	P32119	PRDX2	X	X	X		X
Coagulation factor IX	FA9_HUMAN	P00740	F9	X	X		X	X
Plasma protease C1 inhibitor	IC1_HUMAN	P05155	SERPING1	X		X	X	X
Vitronectin	VTNC_HUMAN	P04004	VTN	X		X	X	X
Histidine-rich glycoprotein	HRG_HUMAN	P04196	HRG	X		X	X	X
Complement component C9	CO9_HUMAN	P02748	C9	X		X	X	X
Transferrin receptor protein 1	TFR1_HUMAN	P02786	TFRC		X	X	X	X
Complement C1q subcomponent subunit C	C1QC_HUMAN	P02747	C1QC		X	X	X	X
Immunoglobulin lambda variable 3-10	LV310_HUMAN	A0A075B6K4	IGLV3-10		X	X	X	X
Immunoglobulin lambda variable 3-9	LV39_HUMAN	A0A075B6K5	IGLV3-9		X	X		X
Galectin-3-binding protein	LG3BP_HUMAN	Q08380	LGALS3BP		X	X	X	X
Immunoglobulin lambda constant 7	IGLC7_HUMAN	A0M8Q6	IGLC7		X	X	X	X
C4b-binding protein beta chain	C4BPB_HUMAN	P20851	C4BPB		X	X	X	X
Pigment epithelium-derived factor	PEDF_HUMAN	P36955	SERPINF1		X	X	X	X
Hemoglobin subunit delta	HBD_HUMAN	P02042	HBD		X	X	X	X
Hemoglobin subunit alpha	HBA_HUMAN	P69905	HBA1		X	X	X	X
Apolipoprotein L1	APOL1_HUMAN	O14791	APOL1	X	X	X		
Fibronectin	FINC_HUMAN	P02751	FN1	X	X		X	
Plasminogen	PLMN_HUMAN	P00747	PLG	X	X		X	
Complement component C8 gamma chain	CO8G_HUMAN	P07360	C8G	X	X		X	
Vitamin K-dependent protein S	PROS_HUMAN	P07225	PROS1	X	X		X	

Fibrinogen alpha chain	FIBA_HUMAN	P02671	FGA	X	X		X	
Nuclear receptor coactivator 6	NCOA6_HUMAN	Q14686	NCOA6	X	X		X	
Hyaluronan-binding protein 2	HABP2_HUMAN	Q14520	HABP2	X	X			X
Cadherin-5	CADH5_HUMAN	P33151	CDH5	X	X			X
IgGFc-binding protein	FCGBP_HUMAN	Q9Y6R7	FCGBP	X	X			X
Lipopolysaccharide-binding protein	LBP_HUMAN	P18428	LBP	X	X			X
Cathelicidin antimicrobial peptide	CAMP_HUMAN	P49913	CAMP	X	X			X
C4b-binding protein alpha chain	C4BPA_HUMAN	P04003	C4BPA	X			X	X
Keratin, type I cytoskeletal 14	K1C14_HUMAN	P02533	KRT14	X			X	X
Beta-2-glycoprotein 1	APOH_HUMAN	P02749	APOH	X			X	X
Sex hormone-binding globulin	SHBG_HUMAN	P04278	SHBG	X			X	X
Haptoglobin	HPT_HUMAN	P00738	HP	X			X	X
Apolipoprotein A-II	APOA2_HUMAN	P02652	APOA2		X	X	X	
Apolipoprotein A-I	APOA1_HUMAN	P02647	APOA1		X	X	X	
Complement C1q subcomponent subunit A	C1QA_HUMAN	P02745	C1QA		X	X	X	
Gelsolin	GELS_HUMAN	P06396	GSN		X	X		X
Complement component C6	CO6_HUMAN	P13671	C6		X	X		X
Immunoglobulin lambda variable 1-40	LV140_HUMAN	P01703	IGLV1-40		X	X		X
Complement C4-A	CO4A_HUMAN	P0C0L4	C4A		X	X		X
Apolipoprotein C-I	APOC1_HUMAN	P02654	APOC1		X	X		X
Immunoglobulin lambda variable 1-47	LV147_HUMAN	P01700	IGLV1-47		X	X		X
Apolipoprotein C-II	APOC2_HUMAN	P02655	APOC2		X	X		X
Immunoglobulin heavy variable 4-31	HV431_HUMAN	P0DP07	IGHV4-31		X	X		X

Kallistatin	KAIN_HUMAN	P29622	SERPINA4		X	X		X
Properdin	PROP_HUMAN	P27918	CFP		X		X	X
Monocyte differentiation antigen CD14	CD14_HUMAN	P08571	CD14		X		X	X
Cholesteryl ester transfer protein	CETP_HUMAN	P11597	CETP		X		X	X
Heparin cofactor 2	HEP2_HUMAN	P05546	SERPIND1		X		X	X
Afamin	AFAM_HUMAN	P43652	AFM		X		X	X
Complement factor H-related protein 4	FHR4_HUMAN	Q92496	CFHR4		X		X	X
Apolipoprotein C-IV	APOC4_HUMAN	P55056	APOC4		X		X	X
Polymeric immunoglobulin receptor	PIGR_HUMAN	P01833	PIGR			X	X	X
Prenylcysteine oxidase 1	PCYOX_HUMAN	Q9UHG3	PCYOX1			X	X	X
Zinc-alpha-2-glycoprotein	ZA2G_HUMAN	P25311	AZGP1			X	X	X
Xaa-Pro dipeptidase	PEPD_HUMAN	P12955	PEPD			X	X	X
Transthyretin	TTHY_HUMAN	P02766	TTR			X	X	X
Keratin, type II cytoskeletal 2 epidermal	K22E_HUMAN	P35908	KRT2			X	X	X
Alpha-2-antiplasmin	A2AP_HUMAN	P08697	SERPINF2	X	X			
Complement factor B	CFAB_HUMAN	P00751	CFB	X	X			
Coagulation factor X	FA10_HUMAN	P00742	F10	X	X			
Collectin-11	COL11_HUMAN	Q9BWP8	COLEC11	X	X			
Beta-Ala-His dipeptidase	CNDP1_HUMAN	Q96KN2	CNDP1	X	X			
Immunoglobulin heavy variable 4-28	HV428_HUMAN	A0A0C4DH34	IGHV4-28	X		X		
Corticosteroid-binding globulin	CBG_HUMAN	P08185	SERPINA	X		X		

			6					
Vitamin K-dependent protein C	PROC_HUMAN	P04070	PROC	X		X		
Carboxypeptidase N catalytic chain	CBPN_HUMAN	P15169	CPN1	X		X		
Immunoglobulin heavy variable 3-72	HV372_HUMAN	A0A0B4J1Y 9	IGHV3- 72	X			X	
Complement C1r subcomponent	C1R_HUMAN	P00736	C1R	X			X	
Carboxypeptidase N subunit	CPN2_HUMAN	P22792	CPN2	X			X	
Ceruloplasmin	CERU_HUMAN	P00450	CP	X			X	
Ficolin-2	FCN2_HUMAN	Q15485	FCN2	X			X	
Alpha-2-macroglobulin	A2MG_HUMAN	P01023	A2M	X			X	
Inter-alpha-trypsin inhibitor heavy chain H3	ITIH3_HUMAN	Q06033	ITIH3	X			X	
Immunoglobulin kappa variable 1-8	KV108_HUMAN	A0A0C4DH 67	IGKV1-8	X			X	
Ficolin-3	FCN3_HUMAN	O75636	FCN3	X			X	
Immunoglobulin heavy variable 7-4-1	HV741_HUMAN	A0A0J9YV Y3	IGHV7-4- 1	X				X
Plasma serine protease inhibitor	IPSP_HUMAN	P05154	SERPINA 5	X				X
Dopamine beta-hydroxylase	DOPO_HUMAN	P09172	DBH	X				X
Hemopexin	HEMO_HUMAN	P02790	HPX	X				X
Serum paraoxonase/arylesterase 1	PON1_HUMAN	P27169	PON1		X	X		
Immunoglobulin kappa variable 3D-15	KVD15_HUMAN	A0A087WS Y6	IGKV3D- 15		X	X		
Immunoglobulin lambda variable 3-19	LV319_HUMAN	P01714	IGLV3- 19		X	X		
Mannose-binding protein C	MBL2_HUMAN	P11226	MBL2		X	X		
Immunoglobulin kappa variable 1-33	KV133_HUMAN	P01594	IGKV1- 33		X	X		

Cholinesterase	CHLE_HUMAN	P06276	BCHE		X	X		
Immunoglobulin lambda variable 7-46	LV746_HUMAN	A0A075B6I9	IGLV7-46		X	X		
Leucine-rich alpha-2-glycoprotein	A2GL_HUMAN	P02750	LRG1		X	X		
Alpha-1-acid glycoprotein 1	A1AG1_HUMAN	P02763	ORM1		X	X		
Immunoglobulin lambda variable 3-25	LV325_HUMAN	P01717	IGLV3-25		X	X		
Alpha-1-antichymotrypsin	AACT_HUMAN	P01011	SERPINA3		X	X		
Tenascin-X	TENX_HUMAN	P22105	TNXB		X	X		
Immunoglobulin kappa variable 6D-21	KVD21_HUMAN	A0A0A0MT36	IGKV6D-21		X	X		
Coagulation factor XIII B chain	F13B_HUMAN	P05160	F13B		X	X		
Extracellular matrix protein 1	ECM1_HUMAN	Q16610	ECM1		X		X	
Thrombospondin-1	TSP1_HUMAN	P07996	THBS1		X		X	
Apolipoprotein M	APOM_HUMAN	O95445	APOM		X		X	
Complement C3	CO3_HUMAN	P01024	C3		X		X	
Haptoglobin-related protein	HPTR_HUMAN	P00739	HPR		X		X	
Alpha-1B-glycoprotein	A1BG_HUMAN	P04217	A1BG		X		X	
Apolipoprotein(a)	APOA_HUMAN	P08519	LPA		X		X	
Inter-alpha-trypsin inhibitor heavy chain H2	ITIH2_HUMAN	P19823	ITIH2		X		X	
Angiotensinogen	ANGT_HUMAN	P01019	AGT		X		X	
Phosphatidylcholine-sterol acyltransferase	LCAT_HUMAN	P04180	LCAT		X		X	
Serum albumin	ALBU_HUMAN	P02768	ALB		X		X	
Insulin-like growth factor-binding protein complex acid labile subunit	ALS_HUMAN	P35858	IGFALS		X		X	
Kininogen-1	KNG1_HUMAN	P01042	KNG1		X		X	

Carbonic anhydrase 1	CAH1_HUMAN	P00915	CA1		X		X	
Complement factor H	CFAH_HUMAN	P08603	CFH		X		X	
Immunoglobulin heavy variable 1-46	HV146_HUMAN	P01743	IGHV1-46		X		X	
Mannan-binding lectin serine protease 2	MASP2_HUMAN	O00187	MASP2		X		X	
Immunoglobulin kappa variable 1-16	KV116_HUMAN	P04430	IGKV1-16		X		X	
Carboxypeptidase B2	CBPB2_HUMAN	Q96IY4	CPB2		X		X	
Inter-alpha-trypsin inhibitor heavy chain H1	ITIH1_HUMAN	P19827	ITIH1		X		X	
Vitamin D-binding protein	VTDB_HUMAN	P02774	GC		X		X	
Complement factor H-related protein 1	FHR1_HUMAN	Q03591	CFHR1		X		X	
Inter-alpha-trypsin inhibitor heavy chain H4	ITIH4_HUMAN	Q14624	ITIH4		X		X	
Proteoglycan 4	PRG4_HUMAN	Q92954	PRG4		X		X	
Immunoglobulin J chain	IGJ_HUMAN	P01591	JCHAIN		X		X	
Complement factor D	CFAD_HUMAN	P00746	CFD		X		X	
Complement C1q subcomponent subunit B	C1QB_HUMAN	P02746	C1QB		X		X	
Alpha-2-HS-glycoprotein	FETUA_HUMAN	P02765	AHSG		X		X	
Immunoglobulin heavy variable 1-2	HV102_HUMAN	P23083	IGHV1-2		X			X
Retinol-binding protein 4	RET4_HUMAN	P02753	RBP4		X			X
Alpha-1-acid glycoprotein 2	A1AG2_HUMAN	P19652	ORM2		X			X
Protein AMBP	AMBP_HUMAN	P02760	AMBP		X			X
Carbonic anhydrase 2	CAH2_HUMAN	P00918	CA2		X			X
Lumican	LUM_HUMAN	P51884	LUM		X			X
Complement factor I	CFAI_HUMAN	P05156	CFI		X			X
Tetranectin	TETN_HUMAN	P05452	CLEC3B		X			X

Complement component C8 alpha chain	CO8A_HUMAN	P07357	C8A			X	X	
Immunoglobulin heavy variable 4-61	HV461_HUMAN	A0A0C4DH41	IGHV4-61			X	X	
Immunoglobulin lambda variable 2-11	LV211_HUMAN	P01706	IGLV2-11			X	X	
Immunoglobulin heavy variable 2-70D	HV70D_HUMAN	A0A0C4DH43	IGHV2-70D			X	X	
Apolipoprotein A-IV	APOA4_HUMAN	P06727	APOA4			X		X
Immunoglobulin kappa variable 1-13	KV113_HUMAN	P0DP09	IGKV1-13			X		X
Immunoglobulin kappa variable 1D-8	KVD08_HUMAN	A0A087WSZ0	IGKV1D-8				X	X
Immunoglobulin kappa variable 2D-29	KVD29_HUMAN	A0A075B6S2	IGKV2D-29				X	X
DDB1- and CUL4-associated factor 12-like protein 1	DC121_HUMAN	Q5VU92	DCAF12L1				X	X
Matrix metalloproteinase-9	MMP9_HUMAN	P14780	MMP9				X	X
Clusterin	CLUS_HUMAN	P10909	CLU				X	X
von Willebrand factor	VWF_HUMAN	P04275	VWF				X	X
Glutathione peroxidase 3	GPX3_HUMAN	P22352	GPX3				X	X
Complement component C7	CO7_HUMAN	P10643	C7				X	X
Keratin, type I cytoskeletal 9	K1C9_HUMAN	P35527	KRT9				X	X
Immunoglobulin heavy variable 1-8	HV108_HUMAN	P0DP01	IGHV1-8				X	X
L-selectin	LYAM1_HUMAN	P14151	SELL				X	X
Fetuin-B	FETUB_HUMAN	Q9UGM5	FETUB				X	X
